# Heparin administration at first medical contact vs immediately before primary percutaneous coronary intervention: the HELP-PCI trial

**DOI:** 10.1093/eurheartj/ehaf481

**Published:** 2025-08-01

**Authors:** Jing Chen, Changwu Xu, Liqiang Qiu, Bin Li, Wei Yao, Hui Wu, Longcai Hu, Guang Xu, Dongmei Zhu, Zhengzai Li, Xiaolin Wu, Chuang Xiao, Bo Liu, Xiuzhen Shen, Zhaowu Deng, Chaogui Zhuo, Huajun Su, Keping Yang, Youen Zhang, Meichun Zhang, Chang Li, Xuexiang Lv, Lifeng Hong, Fan Guo, Xingan Wu, Hao Xu, Mingjian Li, Liqun He, Wenjun Wu, Yuhua Lei, Dongsheng Li, Huoping Li, Shaoze Chen, Hong Bao, Xuguang Xiong, Bo Liu, Guokang Yang, Jinke Chen, Xiao Lan, Qibin Zheng, Guanglong Yang, Mingxi Zhang, Jian Yang, Hong Jiang

**Affiliations:** Department of Cardiology, Renmin Hospital of Wuhan University, Cardiovascular Research Institute of Wuhan University, No. 99. Ziyang Rd., Wuhan, 430060, China; Department of Cardiology, Renmin Hospital of Wuhan University, Cardiovascular Research Institute of Wuhan University, No. 99. Ziyang Rd., Wuhan, 430060, China; Department of Cardiology, Renmin Hospital of Wuhan University, Cardiovascular Research Institute of Wuhan University, No. 99. Ziyang Rd., Wuhan, 430060, China; Department of Cardiology, Xianning Central Hospital, Xianning, China; Department of Cardiology, Suizhou Central Hospital, Suizhou, China; Department of Cardiology, The First College of Clinical Medical Science, Three Gorges University & Yichang Central People's Hospital, Hubei Key Laboratory of Ischemic Cardiovascular Disease, Yichang, China; Department of Cardiology, Tongcheng People's Hospital, Xianning, China; Department of Cardiology, Ezhou Central Hospital, Ezhou, China; Department of Cardiology, Xiantao First People's Hospital, Xiantao, China; Department of Cardiology, Xiantao First People's Hospital, Xiantao, China; Department of Cardiology, Xiangyang Central Hospital, Xiangyang, China; Department of Cardiology, Songzi People's Hospital, Jingzhou, China; Department of Cardiology, People's Hospital of Jingshan, Jingmen, China; Department of Cardiology, Chibi General Hospital, Xianning, China; Department of Cardiology, Jiangxia First People's Hospital, Wuhan, China; Department of Cardiology, Anlu People's Hospital, Xiaogan, China; Department of Cardiology, Caidian People's Hospital, Wuhan, China; Department of Cardiology, Jingzhou Central Hospital, Jingzhou, China; Department of Cardiology, Shiyan People's Hospital, Shiyan, China; Department of Cardiology, Wuhan Puren Hospital, Wuhan, China; Department of Cardiology, Hubei Zhongshan Hospital, Wuhan, China; Department of Cardiology, CR & WISCO General Hospital, Wuhan, China; Department of Cardiology, Wuhan Fifth Hospital, Wuhan, China; Department of Cardiology, Wuhan Fifth Hospital, Wuhan, China; Department of Cardiology, General Hospital of Yangtze River Shipping, Wuhan, China; Department of Cardiology, Guoyao Dongfeng General Hospital, Shiyan, China; Department of Cardiology, Xiangyang Hospital of Traditional Chinese Medicine, Xiangyang, China; Department of Cardiology, Wuhan No. 1 Hospital, Wuhan, China; Department of Cardiology, Xishui People's Hospital, Huanggang, China; Department of Cardiology, The Central Hospital of Enshi Tujia and Miao Autonomous Prefecture, Enshi, China; Department of Cardiology, Wuhan No.3 Hospital, Wuhan, China; Department of Cardiology, Huanggang Central Hospital, Huanggang, China; Department of Cardiology, Huanggang Central Hospital, Huanggang, China; Department of Cardiology, Jiangling People's Hospital, Jingzhou, China; Department of Cardiology, Laohekou First Hospital, Xiangyang, China; Department of Cardiology, Wuhan No.6 Hospital, Wuhan, China; Department of Cardiology, Wuhan Hanyang Hospital, Wuhan, China; Department of Cardiology, Wuxue First People's Hospital, Huanggang, China; Department of Cardiology, Wuxue First People's Hospital, Huanggang, China; Department of Cardiology, Xianning First People's Hospital, Xianning, China; Department of Cardiology, Tianmen First People's Hospital, Tianmen, China; Department of Cardiology, Wuhan Hospital of Traditional Chinese Medicine, Wuhan, China; Department of Cardiology, The First College of Clinical Medical Science, Three Gorges University & Yichang Central People's Hospital, Hubei Key Laboratory of Ischemic Cardiovascular Disease, Yichang, China; Department of Cardiology, Renmin Hospital of Wuhan University, Cardiovascular Research Institute of Wuhan University, No. 99. Ziyang Rd., Wuhan, 430060, China

**Keywords:** myocardial infarction, Unfractionated heparin, Primary percutaneous coronary intervention

## Abstract

**Background and Aims:**

The beneficial effect of pre-treatment with unfractionated heparin (UFH) at first medical contact (FMC) before primary percutaneous coronary intervention (PPCI) in all-comers with ST-elevation myocardial infarction (STEMI) remains uncertain.

**Methods:**

HELP-PCI was an investigator-initiated, randomized controlled trial conducted at 36 clinical centres in China. Patients with STEMI presenting ≤12 h after symptom onset undergoing PPCI were randomly assigned (1:1) to intravenous administration with UFH (100 U/kg) at FMC or in the Cath Lab through a catheter sheath. The primary endpoint was Thrombolysis in Myocardial Infarction flow grade (TFG)-3 of infarct-related artery (IRA) at diagnostic angiography before PPCI. The secondary outcome was complete epicardial and myocardial reperfusion after PPCI and major adverse cardiac and cerebrovascular events (MACCE; defined as the composite of all-cause death, cardiac death, heart failure hospitalizations, re-infarction, stent thrombosis, unplanned revascularization, and stroke) at 12 months. Safety outcome was 30-day Bleeding Academic Research Consortium (BARC) type ≥2 bleeding.

**Results:**

A total of 999 patients with STEMI undergoing PPCI were randomly assigned to receive either UFH administration at FMC (n = 505) or in the Cath Lab (*n* = 494). Pre-treated population at FMC showed a higher frequency of TFG-3 of IRA compared with the Cath Lab group (23.6% vs 17.6%; odds ratio, 1.44; 95% confidence interval, 1.06–1.97; *P* = .02). There were no significant differences in secondary endpoints or in the safety endpoint, including 12-month MACCE, complete epicardial and myocardial reperfusion, and major bleeding.

**Conclusions:**

Pre-treatment with loading-dose UFH at FMC was associated with an improvement of spontaneous reperfusion of IRA without increasing the risk of major bleeding.

## Structured Graphical Abstract

**Structured Graphical Abstract:**
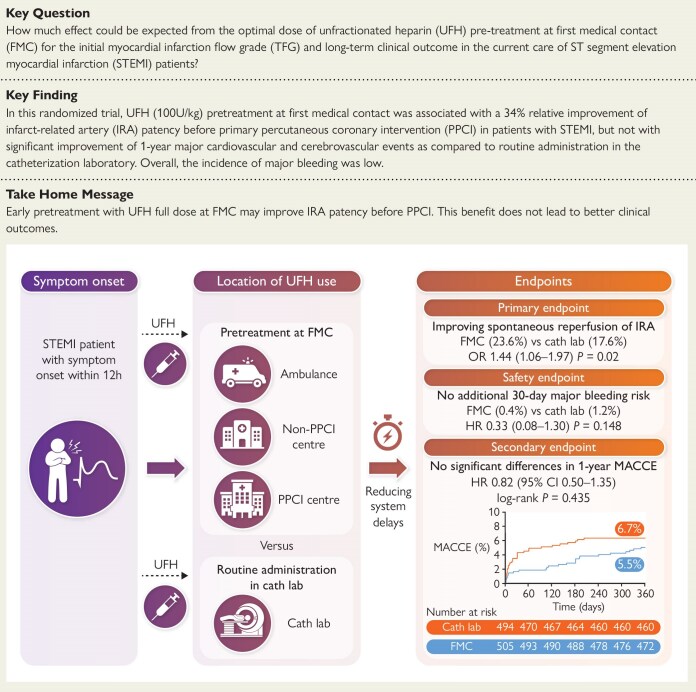
Pre-treatment with a loading dose of UFH at FMC could improve early restoration of IRA and further reduce the system delay in the setting of current STEMI networks. CI, confidence interval; FAS, full analysis set; PPCI, primary percutaneous coronary intervention; FMC, first medical contact; HR, hazard ratio; IRA, infarct-related artery; MACCE, major adverse cardiac and cerebrovascular events; STEMI, ST-elevation myocardial infarction; UFH, unfractionated heparin. UFH pretreatment at FMC in patients with STEMI

**See the editorial comment for this article ‘With a little HELP from heparin at first medical contact before primary percutaneous coronary intervention’, by D. Erlinge and S. Koul, https://doi.org/10.1093/eurheartj/ehaf562.**

## Introduction

Primary percutaneous coronary intervention (PPCI) is the standard reperfusion strategy for patients with ST-elevation myocardial infarction (STEMI), especially when it can be performed within 120 min of initial diagnosis.^1,2^ Although regional networks have been well established worldwide, inherent delays still exist from first medical contact (FMC) transfer to the catheterization laboratory (Cath Lab), during which no specific reperfusion therapy can be administered.^1,2^ Intravenous antithrombotic treatments before PPCI have been tried in the FINESSE trial, but neither early use with abciximab nor half-dose reteplase significantly improved the clinical outcomes in STEMI patients undergoing PPCI, although initial Thrombolysis in Myocardial Infarction (TIMI) flow grade 3 (TFG-3) was increased substantially.^3^ This pharmacologic strategy might increase the risk of major bleeding and mortality, thereby nullifying any potential benefits in terms of clinical outcomes.^3^ Consequently, any treatment that increases infarct-related artery (IRA) patency without increasing complications could be expected to provide a better prognosis.

In addition to aspirin, guidelines recommend using potent P2Y_12_ inhibitors in patients with STEMI under controlled risk of bleeding in regard to more favourable pharmacological properties.^1,2^ However, prehospital administration of ticagrelor did not improve early coronary reperfusion.^4^ Given that the emergency network is now well established, the relatively shorter interval between dual antiplatelet therapy (DAPT) administration at FMC and PPCI may not allow sufficient time for its full benefit to take effect.^4^ Early administration of unfractionated heparin (UFH), commonly used in many national STEMI networks, appears to be both safe and associated with improved TFG before PPCI.^5,6^ A report from the Swedish Coronary Angiography and Angioplasty Registry (SCAAR) indicated that 38% of STEMI patients were indeed receiving UFH pre-treatment.^6^ From a pathophysiological perspective, UFH reaches its maximum effect within minutes, with a half-life of only ∼1 to 2 h after intravenous administration.^6^ These characteristics potentially make UFH a strong candidate for early administration. However, high-quality evidence regarding its efficacy in FMC settings remains limited. To our knowledge, no large randomized clinical trial has been performed to examine whether UFH pre-treatment affects initial TFG and long-term clinical outcomes.

According to guidelines,^1,2^ the recommended UFH dose for initial treatment and PPCI procedures is 70–100 U/kg. The current study aimed to investigate the effects of pre-treatment with UFH at a full dose of 100 U/kg on the restoration of TFG-3 in the IRA of patients with STEMI before PPCI, which, in turn, may be associated with prognosis.

## Methods

### Study design

The HELP-PCI trial was an investigator-initiated, multicentre, open-label, randomized controlled trial (RCT) conducted at 36 clinical centres in 14 cities in China. Patients presenting with STEMI within 12 h of symptom onset undergoing PPCI were randomly assigned to receive UFH (100 U/kg) through intravenous administration either at FMC (in the ambulance, non-PPCI-capable hospital, emergency room) or in the Cath Lab through catheter sheath. The current study complied with the Declaration of Helsinki, and the study protocol was approved by the ethics committee of Renmin Hospital of Wuhan University and each participating site. All patients or their relatives provided written informed consent. The final study protocol and statistical analysis plan are available in Supplementary data online, *Study Protocol*.

### Patient selection

Briefly, patients who met the following STEMI criteria were as follows: (1) age of 18–80 years; (2) STEMI within 12 h of symptom onset (STEMI was defined as ST-segment elevation ≥1 mm in ≥2 contiguous leads (V2–V3 lead elevation ≥2 mm) or new left bundle branch block, or right bundle branch block with ST-segment elevation or Q-wave in leads V1–V3); (3) intended to perform PPCI (expected time interval from FMC to balloon dilation ≤120 min and without considering thrombolysis). Key exclusion criteria were as follows: cardiopulmonary resuscitation, mechanical complication, active bleeding, oral anticoagulation, history of coronary artery bypass grafting, and history of heparin-induced thrombocytopenia.

### Randomization and study procedures

Patients who satisfied the inclusion and exclusion criteria were randomly assigned in a 1:1 ratio to either pre-treatment with UFH at FMC or routine administration in the Cath Lab, using an interactive web-based response system with a stratified block randomization scheme. All of the patients will receive a loading dose of DAPT, comprising 300 mg aspirin and either 180 mg ticagrelor or 300–600 mg clopidogrel at FMC immediately following diagnostic electrocardiogram (ECG). Pre-treated UFH was required to be administered as an initial dose of 100 U/kg intravenous bolus within 10 min after randomization at FMC and should be finished at least before transportation to the Cath Lab. In the Cath Lab group, an initial dose of 100 U/kg UFH was encouraged to be administered through an artery sheath, but the acute dosage was left to the interventional cardiologist’s discretion. In both groups, additional UFH was allowed to be administered during PPCI if the activated clotting time was <225 s or if the physician determined it necessary, with 1000 U UFH given hourly during patient transport.^7^ Premedication with low molecular weight heparin, bivalirudin, glycoprotein IIb/IIIa inhibitor (GPI), or other antithrombotic medications was not allowed in any patient before PPCI. However, provisional GPI use was permitted during or after catheterization at the investigator’s discretion. The PPCI was preferred using the radial artery approach and performed according to standard clinical practice. A 12/18-lead ECG was repeated at 90 min after PPCI. Other medications were provided as recommended in current guidelines.^1,2^ Follow-up was performed 30 days, 6 months, and 12 months after random assignment through office visits or telephone interviews.

### Endpoints and definitions

The primary endpoint was the TFG-3 of IRA at diagnostic angiography before the PPCI procedure. Secondary endpoints were major adverse cardiac and cerebrovascular events (MACCE; the composite of all-cause death, cardiac death, heart failure hospitalizations, myocardial infarction, stent thrombosis, unplanned revascularization, and stroke) and its components at 1 year after randomization. Another secondary endpoint was complete epicardial and myocardial perfusion after PPCI, defined as TFG-3 for epicardial reperfusion and TIMI myocardial perfusion grade (TMPG)-3 for myocardial reperfusion and complete (≥70%) ST-segment resolution (STR) at 90 min after PPCI.^8^ The primary safety endpoint was Bleeding Academic Research Consortium (BARC) type ≥ 2 bleeding 30 days after randomization. All angiographic endpoints and ECG analysis were determined in a blinded fashion by a central core laboratory. *Post hoc* analyses were performed for subsequent outcomes of 30-day MACCE, and the effect of GPI use/hospital of FMC/Q-wave infarction on clinical outcomes. All other analyses were pre-specified in Supplementary data online, *Study Protocol*.

### Statistical analysis

The primary analysis was performed on the full analysis set on an intention-to-treat basis, which included all the patients who underwent randomization and received at least emergent angiography. The clinical outcomes were analysed using a time-to-first-event approach. Based on previous observational studies and similar PPCI RCT studies,^9,10–16^ the sample size was estimated assuming that the incidence of TFG-3 before PPCI and after UFH administration at FMC was 15.5% and 9.2% in the Cath Lab from previous trials. Considering an approximate 10% withdrawal rate, a total sample size of 944 patients (472 in each group) was needed for 80% power with a two-sided alpha of .05 using a χ^2^ test with R software (version 4.2.2; R Core Team). Sensitivity analyses were performed in the per-protocol population (patients who received UFH in full conformance to protocol medication and who met all inclusion criteria and no exclusion criteria) and among patients who underwent PPCI. All subgroup analyses presented were pre-specified.

Continuous data are presented as the mean ± standard deviation or medians with interquartile ranges (IQRs) and compared using a Student’s *t*-test or the Wilcoxon rank-sum test as appropriate. Categorical variables are presented as numbers and percentages, which were compared using the χ² test or Fisher’s exact test. No imputation was used to infer missing values. The primary endpoint was conducted using the Cochran–Mantel–Haenszel method stratified by the study centre and expressed as odds ratio (OR), 95% confidence interval (CI), and *P*-values among the two treatment arms. The association between pre-TFG 3 and UFH-to-wire time was analysed using restricted cubic splines through multivariable logistic regression, with adjustment for age, sex, body mass index, smoking history, diabetes mellitus, admission systolic blood pressure, and Killip class. For the secondary and safety endpoints, time-to-first-event rates were estimated using the Kaplan–Meier method and were compared with the log-rank test. Hazard ratios (HR) and 95% CIs were established from a Cox model. A landmark analysis at 30 days was performed to assess for changes in MACCE risks early vs late in relation to UFH pre-treatment. The endpoint of complete epicardial and myocardial perfusion after PPCI was compared using χ^2^ tests. All statistical tests were two-sided, with *P* < .05 deemed statistically significant. All data analyses were performed using R software (version 4.2.2; R Core Team) and Statistical Package for the Social Sciences software (version 26.0; IBM Crop). The trial was registered with ClinicalTrials.gov (NCT05329155).

## Results

### Patients and procedures

**Figure 1:**
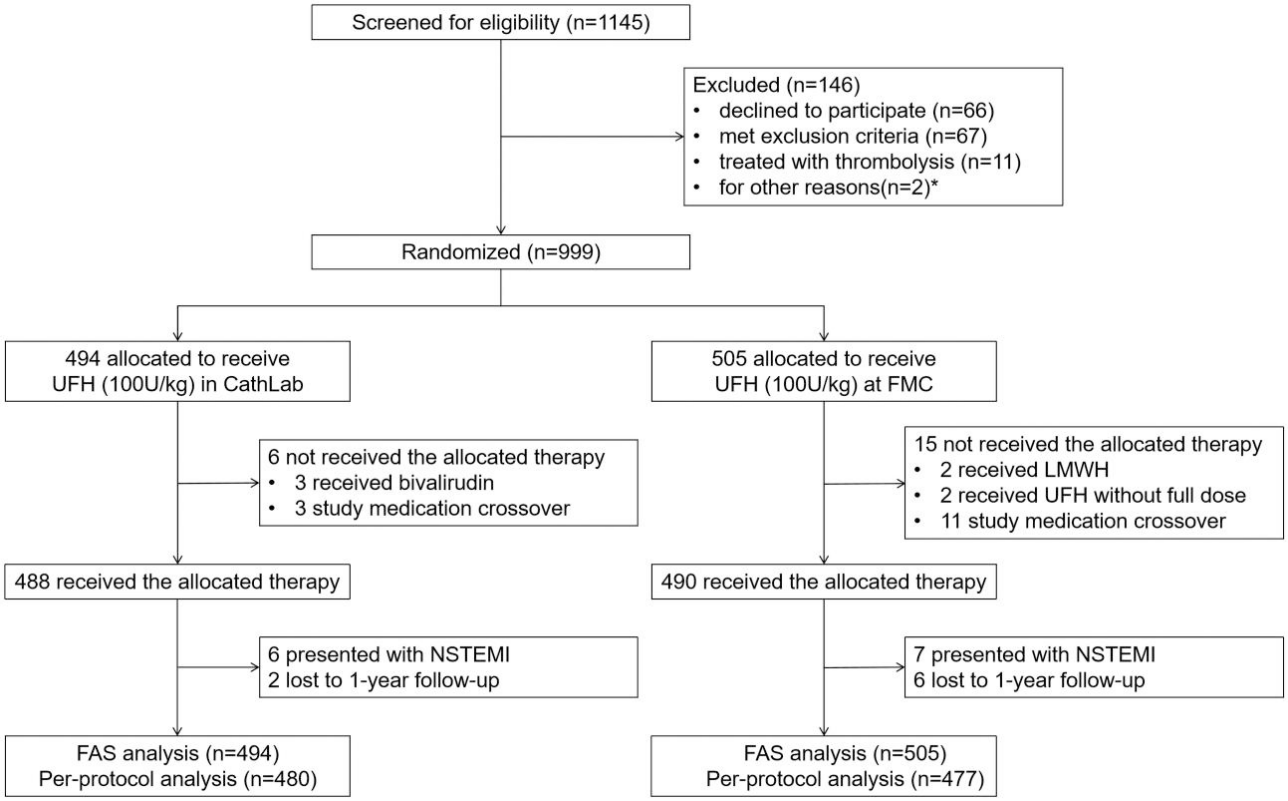
The flowchart of this study. UFH, unfractionated heparin; FMC, first medical contact; LMWH, low molecular weight heparin; NSTEMI, non-ST-elevation myocardial infarction; FAS, full analysis set. *Other reasons for physician request with an unknown reason

Between 20 July 2022, and 20 August 2023, a total of 999 eligible STEMI patients with PPCI from 1145 screened patients were randomly assigned to receive either UFH administration at FMC (*n* = 505) or in Cath Lab (*n* = 494) (*Figure 1*). Baseline characteristics were well balanced between treatment groups (*Table 1*). Study medications and procedural details are depicted in *Table 2*. Coronary angiography was performed using radial artery access in 956 (95.7%) of the 999 patients. A loading dose of DAPT was used in 451 (89.3%) patients in the FMC group and 441 (89.3%) patients in the Cath Lab group. Other medications appeared comparable between the two groups, except that in the FMC group, higher dosages of UFH were found in the angiography subpopulation.

**Table 1:** Baseline characteristics

	Cath Lab (*n* = 494)	FMC (*n* = 505)	*P*-value
**Patient demographics**			
Age (years)	59 (51–68)	60 (51–67)	.888
Sex			.934
Male	405/494 (82.0)	413/505 (81.8)	
Female	89/494 (18.0)	92/505 (18.2)	
BMI (kg/m^2^)	23.7 (22.0–25.8)	23.7 (22.0–25.9)	.909
**Medical history**			
Former or current smoking	216/493 (43.8)	220/505 (43.6)	.937
Diabetes	102/493 (20.7)	116/505 (23.0)	.383
Previous MI	30/493 (6.1)	22/505 (4.4)	.219
Previous stroke	45/493 (9.1)	33/505 (6.5)	.127
Peripheral artery disease	18/493 (3.7)	22/505 (4.4)	.570
**Haemodynamics on admission**			
HR (bpm)	79 (68–88)	76 (67–88)	.194
SBP (mmHg)	125 (110–140)	125 (113–140)	.344
DBP (mmHg)	78 (70–88)	80 (70–90)	.140
Killip class			.453
I	385/494 (77.9)	407/505 (80.6)	
II	54/494 (10.9)	44/505 (8.7)	
III	21/494 (4.3)	26/505 (5.1)	
IV	34/494 (6.9)	28/505 (5.5)	
Non-Q-wave MI^a^	343/494 (69.4)	331/504 (65.7)	.205
Missing data	-	1/505 (0.2)	
**Infarct-related wall**			.917
Anterior	240/494 (48.6)	247/505 (48.9)	
Non-Anterior	254/494 (51.4)	258/505 (51.1)	
**Laboratory data**			
Hb (g/L)	140 (127–151)	140 (128–151)	.840
Anaemia^b^	113/491 (23.0)	110/501 (22.0)	.690
Missing data	3/494 (0.6)	4/505 (0.8)	
NLR	6.6 (3.3–10.6)	6.8 (3.7–10.8)	.134
Missing data	6/494 (1.2)	7/505 (1.4)	
Platelet (10^9^/L)	219 (182–267)	218 (177–265)	.670
Missing data	3/494 (0.6)	4/505 (0.8)	
eGFR (mL/min/1.73 m^2^)^c^	97.2 (80.8–118.9)	96.1 (78.3–117.2)	.220
<60 mL/min/1.73 m^2^	37/492 (7.5)	44/501 (8.8)	.468
Missing data	2/494 (0.4)	4/505 (0.8)	
Creatinine (μmol/L)	73.7 (61.0–85.9)	74.2 (62.6–89.0)	.301
Missing data	2/494 (0.4)	4/505 (0.8)	

Data are presented as median (interquartile range) and n/N (%). FMC, first medical contact; BMI, body mass index; MI, myocardial infarction; HR, heart rate; SBP, systolic blood pressure; DBP, diastolic blood pressure; HB, haemoglobin; NLR, neutrophil-to-lymphocyte ratio; eGFR, estimated glomerular filtration rate.

^a^Diagnostic criteria for pathological Q waves: Duration (Width): Q-wave duration ≥ 40 ms or amplitude (Depth): Q-wave depth > 25% of the R wave amplitude in the same lead, lead characteristics defined as any Q-wave in leads V1–V3 (normally Q waves are absent in these leads) or abnormal Q waves in two contiguous limb leads (e.g. inferior leads II, III, aVF; or lateral leads I, aVL, V5–V6) or an isolated Q-wave in lead III may be a normal variant, but becomes pathological if accompanied by abnormal Q waves in leads II or aVF.

^b^Defined as a haemoglobin concentration <130 g/L in men and <120 g/L in women.

^c^Calculated by the formula: 186× (creatinine [mg/dL])^−1·154^ × (age)^−0·203^ × (0·742 if female).

**Table 2:** Medications and procedural results

	Cath Lab (n = 494)	FMC (n = 505)	*P*-value
**Study medication**			
UFH			
Total dose of UFH during PPCI, U^a^	7000 (6000–7500)	7000 (6000–8000)	.017
PCI	7000 (6000–7500)	7000 (6000–8000)	.063
SCA	3000 (3000–6125)	6000 (5125–6500)	＜.001
DAPT at FMC			
Aspirin with full dose^b^	491/494 (99.4)	500/505 (99.0)	.497
P2Y12 receptor inhibitor with full dose^c^	441/494 (89.3)	451/505 (89.3)	.985
Clopidogerel	21/494 (4.3)	19/505 (3.8)	.694
Ticagrelor	420/494 (85.0)	432/505 (85.5)	.815
GPI use			.517
During PPCI	32/494 (6.5)	32/505 (6.3)	
After PPCI	188/494 (38.1)	210/505 (41.6)	
Opiate-treatment			.108
Before PPCI	10/494 (2.0)	6/505 (1.2)	
During PPCI	22/494 (4.5)	33/505 (6.5)	
After PPCI	59/494 (11.9)	43/505 (8.5)	
**Procedural characteristics**			
Invasive procedural			.818
Transradial	472/494 (95.5)	484/505 (95.8)	
Transfemoral	22/494 (4.5)	21/505 (4.2)	
Infarct-related artery			.426
LM	3/494 (0.6)	1/505 (0.2)	
LAD	239/494 (48.4)	249/505 (49.3)	
LCX	55/494 (11.1)	44/505 (8.7)	
RCA	197/494 (39.9)	211/505 (41.8)	
Coronary artery disease			.184
Single-vessel disease	256/494 (51.8)	274/505 (54.3)	
Double-vessel disease	153/494 (31.0)	136/505 (26.9)	
Three-vessel disease	63/494 (12.8)	80/505 (15.8)	
Complete revascularization	267/494 (54.0)	277/505 (54.9)	.799
PCI	460/494 (93.1)	473/505 (93.7)	.728
DES Implantation	349/494 (70.6)	365/505 (72.3)	.569
Number of stents	1.41 ± .61	1.39 ± 0.61	.656
Total length of stent (mm)	33.3 ± 16.8	33.4 ± 16.6	.734
PTCA	111/494 (22.5)	108/505 (21.4)	.679
DCB	36/494 (7.3)	31/505 (6.1)	.468
Non-drug coat	75/494 (15.2)	77/505 (15.2)	.977
Thrombus aspiration	72/494 (14.6)	60/505 (11.9)	.209
Thrombus grade			.241
0	28/494 (5.7)	30/505 (5.9)	
1	13/494 (2.6)	23/505 (4.6)	
2	26/494 (5.3)	33/505 (6.5)	
3	41/494 (8.3)	44/505 (8.7)	
4	59/494 (11.9)	75/505 (14.9)	
5	327/494 (66.2)	300/505 (59.4)	
Pre-PCI TFG			.018
0	335/494 (67.8)	307/505 (60.8)	
1	21/494 (4.3)	35/505 (6.9)	
2	51/494 (10.3)	44/505 (8.7)	
3	87/494 (17.6)	119/505 (23.6)	
Post-PCI TFG			.832
0	4/494 (0.8)	5/505 (1.0)	
1	6/494 (1.2)	4/505 (0.8)	
2	20/494 (4.0)	17/505 (3.4)	
3	464/494 (93.9)	479/505 (94.9)	
Pre-PCI TMPG			.349
0	356/494 (72.1)	342/505 (67.7)	
1	26/494 (5.3)	25/505 (5.0)	
2	35/494 (7.1)	38/505 (7.5)	
3	77/494 (15.6)	100/505 (19.8)	
Post-PCI TMPG			.437
0	6/494 (1.2)	7/505 (1.4)	
1	7/494 (1.4)	4/505 (0.8)	
2	31/494 (6.3)	43/505 (8.5)	
3	450/494 (91.1)	451/505 (89.3)	
IABP use	22/494 (4.5)	17/505 (3.4)	.375
**Medication at discharge**			
DAPT	399/494 (80.8)	422/505 (83.6)	.248
Statin	470/494 (95.1)	477/505 (94.5)	.625
β Receptor inhibitor	360/494 (72.9)	342/505 (67.7)	.075
ACEI/ARB/ARNI	271/494 (54.9)	283/505 (56.0)	.707

Data are presented as median (interquartile range) and n/N (%), or mean ± SD. UFH, unfractionated heparin; PPCI, primary percutaneous coronary intervention; SCA, selective coronary angiography; FMC, first medical contact; DAPT, dual antiplatelet therapy; GPI, glycoprotein IIb/IIIa inhibitor; LAD, left anterior descending artery; LCX, left circumflex artery; RCA, right coronary artery; LM, left main; DES, drug-eluting stent; PTCA, percutaneous transluminal coronary angioplasty; DCB, drug-coated balloon; TFG, Thrombolysis in Myocardial Infarction flow grade; TMPG, TIMI myocardial perfusion grade; IABP, intra-aortic balloon pump; ACEI, angiotensin-converting enzyme inhibitor; ARB, angiotensin receptor inhibitor; ARNI, angiotensin receptor-neprilysin inhibitor.

^a^DAPT was not taken before PCI in 4 patients (2 in Cath Lab arm, 2 in FMC arm).

^b^Aspirin with full dose was defined as 300 mg.

^c^P2Y12 receptor inhibitor with full dose was defined as clopidogrel of 600 mg or ticagrelor of 180 mg.

About 75.3% of patients arrived directly at a PPCI-capable hospital by ambulance (*n* = 41) or non-EMS transportation (*n* = 711), and 24.7% of patients were transferred from a non-PPCI-capable hospital. Total ischaemic time and randomization intervals were similar between the two groups (*Table 3*). The median time between symptom onset and wire time was 3.58 h (IQR: 2.34–5.48) in the FMC group and 3.36 h (IQR: 2.30–5.09) in the Cath Lab group, respectively. The DAPT was administered immediately at FMC with an average time of 14 min in these two groups. Pre-treatment with UFH achieved significantly shorter FMC to UFH intervals [35 min (IQR: 22–63)] than those administered in Cath Lab [71 min (IQR: 47–118); *P* < .001], especially in those patients who were transferred from a non-PPCI-capable hospital. The average time interval from UFH administration to wire time was longer in the FMC group compared with the Cath Lab group.

**Table 3:** Time intervals for primary PCI

	Cath Lab (*n* = 494)	FMC (*n* = 505)	*P*-value
Case			.429
PPCI-capable hospital	363/494 (73.5)	389/505 (77.0)	
Ambulance	20/494 (4.0)	21/505 (4.2)	
Non-EMS transportation	343/494 (69.4)	368/505 (72.9)	
Non-PPCI-capable hospital	131 /494 (26.5)	116/505 (23.0)	
Symptom to wire time (h)	3.36 (2.30–5.09)	3.58 (2.34–5.48)	.239
Symptom to randomization time (h)	2.55 (1.50–4.27)	2.68 (1.54–4.47)	.574
FMC to wire time (h)	1.38 (0.97–2.15)	1.33 (0.98–1.98)	.436
Door to wire time (h)	1.03 (0.78–1.40)	1.08 (0.80–1.41)	.542
Symptom to DAPT time (h)^a^	2.12 (1.18–3.91)	2.42 (1.28–4.01)	.121
FMC to DAPT time (min)^a^	14 (8–31)	14 (9–27)	.917
DAPT to wire time (min)^a^	62 (44–95)	59 (43–86)	.200
Symptom to PCI-capable hospital time (h)	2.11 (1.05–4.00)	2.42 (1.23–4.08)	.818
≤ 6	427/494 (86.4)	439/505 (86.9)	
> 6	67/494 (13.6)	66/505 (13.1)	
Symptom to UFH time (h)	3.10 (2.05–4.90)	2.85 (1.67–4.59)	.002
FMC to UFH time (min)	71 (47–118)	35 (22–63)	< .001
PPCI-capable hospital	57 (43–87)	29 (19–48)	< .001
Ambulance	60 (38–116)	32 (21–64)	.012
Non-EMS transportation	57 (43–86)	29 (19–47)	< .001
Non-PPCI-capable hospital	125 (93–161)	79 (53–120)	< .001
Randomization to UFH time (min)	31 (20–44)	5 (2–10)	< .001
PPCI-capable hospital	29 (19–41)	4 (2–8)	< .001
Ambulance	23 (16–37)	6 (3–9)	< .001
Non-EMS transportation	29 (19–41)	4 (2–8)	< .001
Non-PPCI-capable hospital	36 (24–55)	8 (3–22)	< .001
UFH-to-wire time (min)	12 (8–17)	37 (26–54)	< .001
PPCI-capable hospital	11 (7–16)	38 (25–51)	< .001
Ambulance	12 (10–18)	37 (25–48)	< .001
Non-EMS transportation	11 (6–16)	38 (25–52)	< .001
Non-PPCI-capable hospital	11 (7–18)	34 (24–65)	< .001

Data are presented as median (interquartile range) and *n* (%). UFH, unfractionated heparin; PPCI, primary percutaneous coronary intervention; DAPT, dual antiplatelet therapy; FMC, first medical contact; PPCI, primary percutaneous coronary intervention.

^a^DAPT was not taken before PCI in four patients (two in the Cath Lab arm, two in the FMC arm).

### Primary endpoint

**Figure 2:**
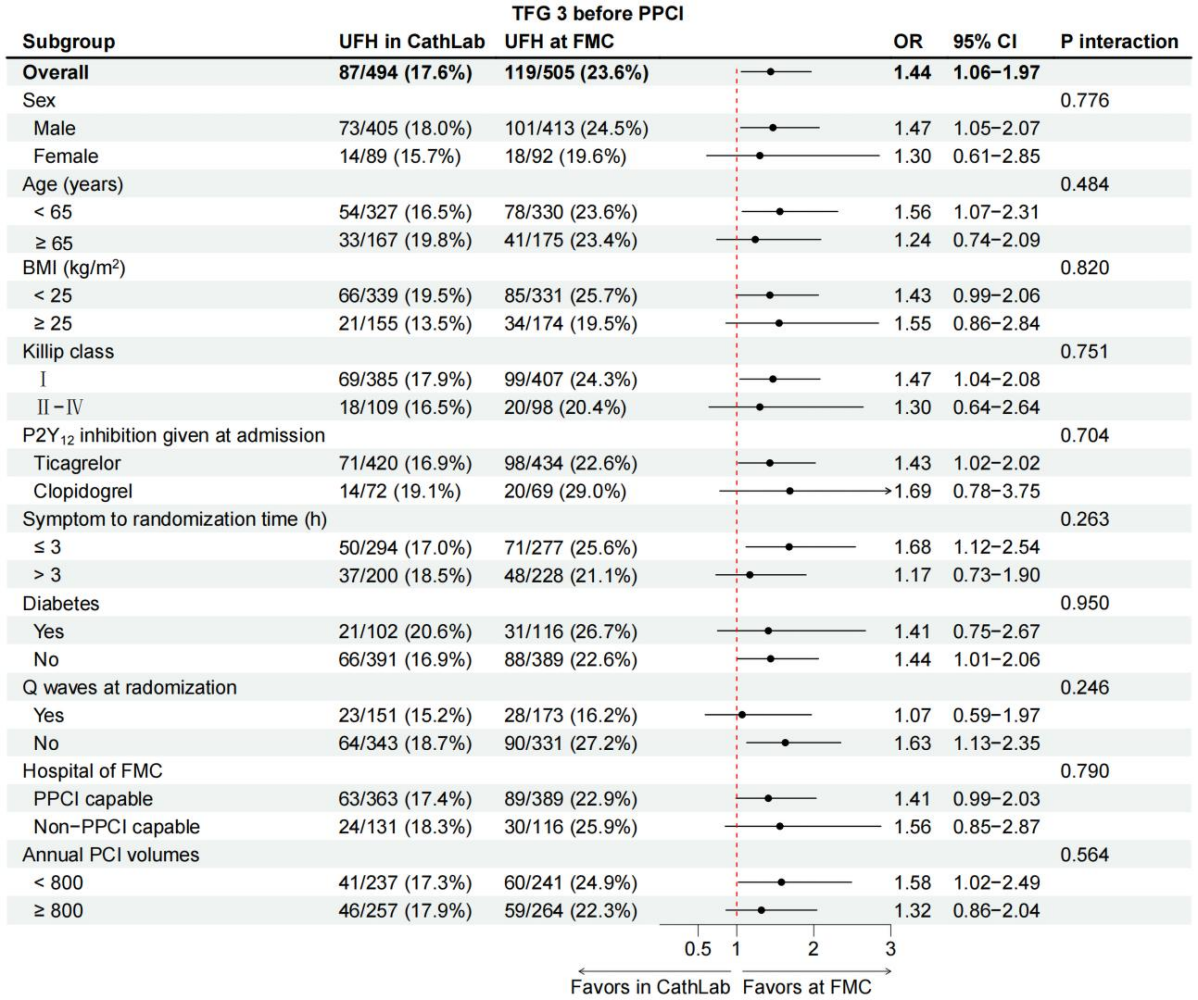
Subgroup analyses of the primary endpoint among the two treatment arms in FAS. FAS, full analysis set; BMI, body mass index; CI, confidence interval; FMC, first medical contact; PCI, percutaneous coronary intervention; OR, odds ratio

A significant difference was observed between UFH treatment at FMC and in Cath Lab regarding the proportion of patients achieving a TFG-3 in the IRA at initial angiography (23.6% in FMC group vs 17.6% in Cath Lab group; OR 1.44; 95% CI 1.06–1.97; *P* = .02) (*Figure 2*). A time-dependent association was found between UFH-wire time interval and initial TFG-3 in the UFH pre-treatment population involved in this study (*n* = 486) after *post hoc* analysis [adjusted OR, 1.05 per 5-min increase for UFH to wire; 95% CI, 1.02–1.09; *P* = .003 (see Supplementary data online, *Figure S1*)]. Further subgroup analyses indicated consistent trends in IRA patency (*Figure 2*). The principal results were similar in the per-protocol population (*n* = 957) and among patients who underwent PCI (*n* = 933) (see Supplementary data online, *Figure S2-S3*).

### Clinical and safety outcomes

**Figure 3:**
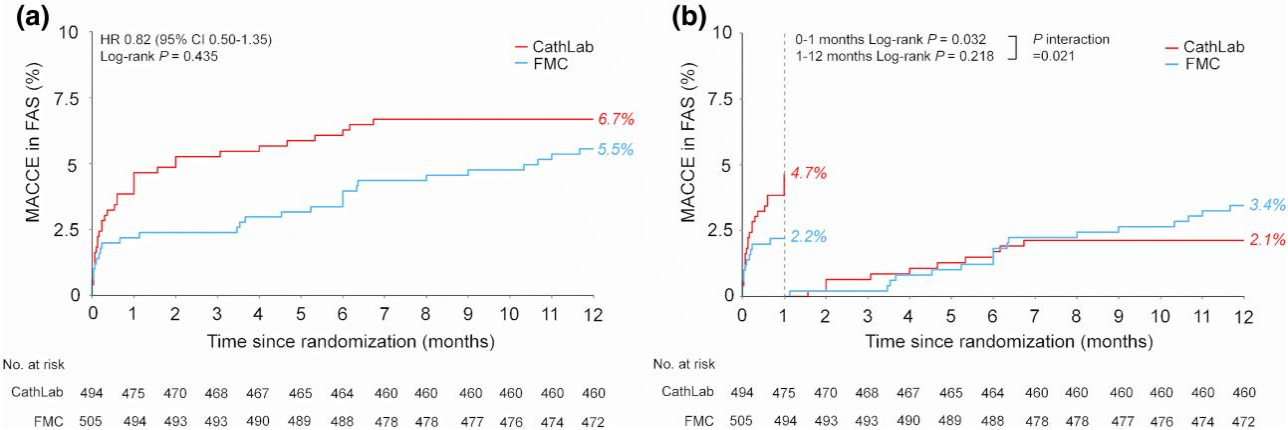
(*A*) Kaplan–Meier curves for the 1-year MACCE in FAS. *(B*) Time-to-event curves for MACCE with a landmark set at 30 days in FAS. FAS, full analysis set; CI, confidence interval; FMC, first medical contact; MACCE, major adverse cardiac and cerebrovascular events

Follow-up data at 1 year were available in 991 (99.2%) randomized patients. The 12-month MACCE occurred in 28 patients in the UFH administration at FMC group and 33 patients in Cath Lab group (Kaplan–Meier rate: 5.5% vs 6.7%; HR, 0.82 [95% CI, 0.50–1.35]; *P* = .44) with non-significant differences in the frequency of the individual components of the combined endpoint (*Figure 3* and *Table 4*). Consistent results were obtained across all examined subgroups for 12-month MACCE with no significant interaction (see Supplementary data online, *Figure S5*).

**Table 4:** Clinical outcomes in FAS

Outcomes	Cath Lab (n = 494)	FMC (n = 505)	HR (95% CI)	*P*-value
**At 30 days**				
MACCE	23/494 (4.7)	11/505 (2.2)	0.47 (0.24–0.91)	.032
Total death	14/494 (2.8)	10/505 (2.0)	0.70 (0.31–1.55)	.378
Cardiac death	14/494 (2.8)	10/505 (2.0)	0.70 (0.31–1.55)	.378
Heart failure hospitalization	5/494 (1.0)	0/505 (0.0)	-	.023
Re-infarction	2/494 (0.4)	0/505 (0.0)	-	.152
Stent thrombus	1/494 (0.2)	0/505 (0.0)	-	.311
Unplanned revascularization	3/494 (0.6)	0/505 (0.0)	-	.080
Stroke	1/494 (0.2)	1/505 (0.2)	0.97 (0.06–15.47)	.981
**At 180 days**				
MACCE	31/494 (6.3)	20/505 (4.0)	0.62 (0.36–1.08)	.095
Total death	15/494 (3.0)	14/505 (2.8)	0.91 (0.44–1.88)	.789
Cardiac death	15/494 (3.0)	14/505 (2.8)	0.91 (0.44–1.88)	.789
Heart failure hospitalization	9/494 (1.8)	3/505 (0.6)	0.32 (0.10–0.99)	.070
Re-infarction	2/494 (0.4)	0/505 (0.0)	-	.152
Stent thrombus	1/494 (0.2)	0/505 (0.0)	-	.311
Unplanned revascularization	5/494 (1.0)	1/505 (0.2)	0.19 (0.04–0.96)	.093
Stroke	2/494 (0.4)	2/505 (0.4)	0.96 (0.14–6.83)	.967
**At 1 year**				
MACCE	33/494 (6.7)	28/505 (5.5)	0.82 (0.50–1.35)	.435
Total death	15/494 (3.0)	20/505 (4.0)	1.29 (0.67–2.51)	.452
Cardiac death	15/494 (3.0)	19/505 (3.8)	1.23 (0.63–2.41)	.552
Heart failure hospitalization	10/494 (2.0)	4/505 (0.8)	0.38 (0.13–1.09)	.092
Re-infarction	2/494 (0.4)	0/505 (0.0)	-	.152
Stent thrombus	1/494 (0.2)	0/505 (0.0)	-	.311
Unplanned revascularization	6/494 (1.2)	1/505 (0.2)	0.16 (0.04–0.71)	.053
Stroke	2/494 (0.4)	4/505 (0.8)	1.92 (0.39–9.54)	.443

Data are presented as *n*/*N* (%).

FAS, full analysis set; CI, confidence interval; FMC, first medical contact; HR, hazard ratio; MACCE, major adverse cardiac and cerebrovascular event.

Interestingly, in the landmark analysis from 0 to 1 month, Kaplan–Meier estimates of MACCE were 2.2% in FMC group vs 4.7% in Cath Lab group (log-rank *P* = .03), whereas no differences in risk compari­sons were observed between groups from 1 to 12 months (log-rank *P* = .22) (*Figure 3*). Furthermore, in a *post hoc* analysis with 30-day MACCE, patients assigned to the UFH pre-treatment group had a lower rate of MACCE (2.2% vs 4.7%; HR, 0.47; 95% CI, 0.24–0.91; *P* = .03) due to a lower rate of heart failure hospitalization (see Supplementary data online, *Figure S4* and *Table 4*). A similar tendency was observed in the 11 subgroups at 30 days after randomization (see Supplementary data online, *Figure S4*). The BARC type 2–5 bleeding events were equal in both groups (FMC vs Cath Lab at 30 days, 0.4% vs 1.2%; FMC vs Cath Lab at 1 year, 1.4% vs 2.0%; all non-significant, *P* > .05; *Table 5*). The principal results were similar in PP and PPCI populations (see Supplementary data online, *Figure S4-S7* and Supplementary data online, *Table S1-S4*).

**Table 5:** Safe endpoints in FAS

	Cath Lab (*n* = 494)	FMC (*n* = 505)	HR (95% CI)	*P*-value
**At 30 days**				
BARC type 2–5 bleeding	6/494 (1.2)	2/505 (0.4)	0.33 (0.08–1.30)	.148
Access-site-related	0/494 (0.0)	0/505 (0.0)	-	-
Non-access-site-related	6/494 (1.2)	2/505 (0.4)	0.33 (0.08–1.3)	.148
Gastrointestinal	4/494 (0.8)	1/505 (0.2)	0.24 (0.04–1.41)	.171
Intracranial	1/494 (0.2)	1/505 (0.2)	0.98 (0.06–15.64)	.988
Other	1/494 (0.2)	0/505 (0.0)	-	.311
**At 180 days**				
BARC type 2–5 bleeding	10/494 (2.0)	7/505 (1.4)	0.68 (0.26–1.76)	.434
Access-site-related	0/494 (0.0)	0/505 (0.0)	-	-
Non-access-site-related	10/494 (2.0)	7/505 (1.4)	0.68 (0.26–1.76)	.434
Gastrointestinal	6/494 (1.2)	4/505 (0.8)	0.65 (0.19–2.24)	.501
Intracranial	1/494 (0.2)	3/505 (0.6)	2.92 (0.41–20.75)	.330
Other	3/494 (0.6)	0/505 (0.0)	-	.079
**At 1 year**				
BARC type 2–5 bleeding	10/494 (2.0)	7/505 (1.4)	0.68 (0.26–1.76)	.434
Access-site-related	0/494 (0.0)	0/505 (0.0)	-	-
Non-access-site-related	10/494 (2.0)	7/505 (1.4)	0.68 (0.26–1.76)	.434
Gastrointestinal	6/494 (1.2)	4/505 (0.8)	0.65 (0.19–2.24)	.501
Intracranial	1/494 (0.2)	3/505 (0.6)	2.92 (0.41–20.75)	.330
Other	3/494 (0.6)	0/505 (0.0)	-	.079

Data are presented are *n*/*N* (%).

FAS, full analysis set; FMC, first medical contact; HR, hazard ratio; CI, confidence interval; BARC, Bleeding Academic Research Consortium.

### Epicardial and myocardial reperfusion

**Figure 4:**
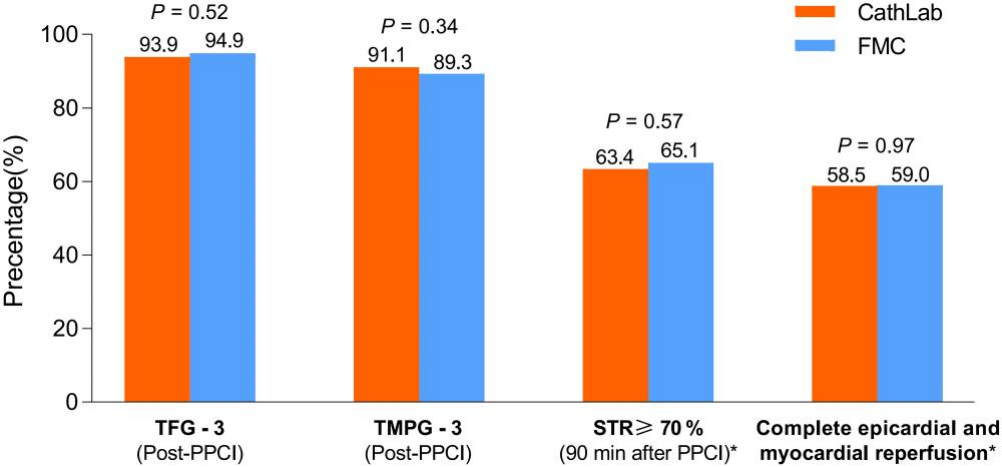
Epicardial and myocardial reperfusion post-PPCI procedure among the 2 treatment arms in FAS. FAS, full analysis set; FMC, first medical contact; PPCI, primary percutaneous coronary intervention; TIMI, thrombolysis in myocardial infarction; TMPG, TIMI myocardial perfusion grade; STR, ST-segment resolution; *23 missing electrocardiogram data in FAS (8 in Cath Lab group and 15 in FMC group)

Non-significant differences occurred in the rate of complete epicardial and myocardial reperfusion between FMC group and Cath Lab group (59.0% vs 58.5%, *P* = .97), defined as TFG-3 after PCI (94.9% vs 93.9%, *P* = .52), TMPG-3 (89.3% vs 91.1%, *P* = .34), and STR ≥ 70% (65.1% vs 63.4%, *P* = .57) (*Figure 4* and Supplementary data online, *Figure S8-S9*).

## Discussion

To our knowledge, HELP-PCI was the largest RCT to compare the efficacy and safety of administration with 100 U/kg UFH at FMC vs in the Cath Lab for STEMI patients undergoing PPCI. The primary findings of the current study are as follows: (i) UFH pre-treatment was associated with a 34.1% relative improvement of IRA patency before PPCI without major bleeding events in the current era of STEMI treatment; (ii) no significant difference was observed in the rate of 1-year MACCE and complete epicardial and myocardial reperfusion regardless of the treatment with UFH at FMC or in Cath Lab (*Structured Graphical Abstract*).

Before this trial, only ∼12 observational studies and one small RCT (conducted in the late 1990s) were performed, with inconsistent results regarding the early restoration of IRA.^6^,^7^,^9–19^ An analysis of outcomes in 7000 STEMI patients from a *post hoc* analysis of the TASTE study demonstrated increased TFG-3 and less intracoronary thrombus burden in those receiving heparin prior to PPCI,^17^ however, this benefit was not confirmed in the single-centre HEAP randomized trial.^11^ The reasons for this difference might relate to total ischaemic time and non-fixed UFH dose. In the current study, early pre-treatment with UFH helped to achieve a 34.1% relative improvement of spontaneous reperfusion before PPCI even within a shorter average time of 37 min from administration of UFH at FMC to PPCI. To our knowledge, early UFH therapy within the ﬁrst 1 or 2 h of symptom onset may be more effective,^13^ as the thrombus is less organized and predominantly non-red during this period.^16^ Overall, the HELP-PCI trial represents good practice with the average FMC to wire time of 1.35 h. These ﬁndings lead us to speculate that shorter symptom onset-to-balloon time might determine the optimal effect of UFH pre-treatment.^4,17^ Another hypothesized mechanism for these findings may be explained by complete antithrombotic pre-treatment with a therapeutic dose of UFH and possible pharmacological synergism with adequate antiplatelet therapy.^10^ Non-full dose of UFH (5000 U or 60 U/kg), used in most of the observational studies, is not likely to have a sufficient anticoagulant effect due to the subtherapeutic activated clotting time.^20^

Contrary to expectations, observed beneﬁts in initial IRA patency did not improve myocardial reperfusion and reduce the rate of MACCE after 1-year follow-up in the current study. Regarding mortality, a statistically non-significant difference between the groups was found in the current study and the other three studies,^7,15,17^ whereas a favour of UFH pre-treatment was reported in another five studies.^6,12–14,18^ Several considerations are relevant for these diverging results, including heterogeneity of the trial population, anticipated effect with different antithrombotic treatments, deficiencies in registry trial conduct, and the expected and actual statistical power. Moreover, the TFG before PPCI as the primary endpoint is a surrogate marker of myocardial reperfusion and might lack enough statistical power for clinical outcomes in our study. Another interesting finding is that the clinical benefit of UFH pre-treatment was mainly observed within the first 1 month revealed by *post hoc* analyses and landmark analysis. These potential benefits at 30 days might be offset by the presence of a late catch-up of MACCE in both 6 and 12 months. It is indeed implausible that a single dose of UFH, which has a short therapeutic effect, could be responsible for such a long-term clinical outcome. Although IRA patency influences salvaging myocardium, the prognosis at a long-term follow-up is probably affected by other interventions. For instance, no additional information was available about the future full revascularization and optimum medical therapy in both groups at follow-up in the current study.

The ideal antithrombotic strategy aims to enhance early IRA patency while minimizing the risk of both ischaemic and haemorrhagic complications during PPCI procedure. Consistent with the therapeutic concept, the large-scale BRIGHT-4 trial have confirmed that bivalirudin with a post-PCI high-dose infusion reduces all-cause mortality and major bleeding compared with heparin monotherapy in patients with STEMI undergoing PPCI.^21^ This benefit seems to persist in the EUROMAX trial by prehospital initiation of bivalirudin treatment compared to heparin plus GPI use in most patients.^22^ Notably, we did not find a significantly increased risk of major bleeding associated with UFH pre-treatment in the HELP-PCI trial, and even more, the rate of major bleeding was indeed lower in both treatment groups in HELP-PCI than in EUROMAX and BRIGHT-4.^21,22^ The reasons for this difference might be arising from a higher use of radial artery access, prohibited use of GPI before PPCI, diverse population traits, or a combination of these factors. It has been reported that UFH pre-treatment was associated with an increased risk of bleeding for patients undergoing non-radial access, age over 75 years old, or low weight.^23^ One may speculate that HELP-PCI trial contains only ∼8.8% of elderly or underweight patients, whereas most of the included population is low to medium-risk individuals, notably less prone to bleeding complications. Radial access is one of the other contributors to reducing life-threatening bleeding complications.^24^ In the current study, 95.7% of procedures were performed by radial access, and no puncture-related major bleeds were observed in the two arms. According to these data, the low risk of bleeding rate suggests this practice is safe in this setting.

The current study has inherent limitations. First, prasugrel was not available in China and was therefore not used in our study. Since ISAR-REACT 5 trial demonstrated no differences in the rates of early IRA patency with STEMI assigned to prasugrel or ticagrelor,^25^ we believe similar results of UFH pre-treatment would be observed in patients treated with prasugrel. Second, GPI in combination with DAPT might break the balance between thrombotic and bleeding risk, thus affecting clinical benefits. However, routine use of GPI did not increase major bleeding in the ATLANTIC trial,^26^ our study further indicated a similar tendency for endpoints in subpopulations receiving GPI or non-GPI use with no significant interaction. Third, although morphine administration delayed onset of action of ticagrelor in the PRIVATE-ATLANTIC study,^27^ the impact of morphine might be minimal in our study due to the short transfer delay and the lower estimated usage rate according to the data on morphine use during and after PPCI (∼1.60%). Fourth, any myocardial damage and coronary microvascular dysfunction must be obtained with more accurate cardiac magnetic resonance data to detect the potential reperfusion benefit with UFH pre-treatment. Finally, given that the sample size estimate for the current study was calculated based on IRA patency, the low rates of MACCE meant that the study was underpowered to detect any potential difference between groups in these clinical outcomes. Therefore, a larger study is needed to evaluate the clinically relevant degrees of risk reduction with UFH pre-treatment in patients with STEMI.

In conclusion, early pre-treatment with full-dose UFH (100 U/kg) at FMC could achieve a greater pre-PPCI TIMI flow grade in the IRA without increased bleeding risk or ischaemic events in STEMI patients undergoing PPCI. The initiation of UFH treatment at FMC could be allowed under the currently efficient organization of the STEMI networks. Further studies are warranted to assess whether the TIMI flow benefit provides additional protection for clinical outcomes.

## Supplementary Material

ehaf481_Supplementary_Data

## Declarations

### Data Availability

The deidentified individual data underlying this article are available from the corresponding author on reasonable request via email.

### Pre-registered Clinical Trial Number

The trial was registered with ClinicalTrials.gov (NCT05329155).
