# Supplementary material for: Heparin administration at first medical contact vs immediately before primary percutaneous coronary intervention: the HELP-PCI trial
Source: Eur Heart J. 2025 Aug 1;46(39):3888–901. doi: 10.1093/eurheartj/ehaf481 (PMC12517749; doi:10.1093/eurheartj/ehaf481)
Supplement: ehaf481_Supplementary_Data [file ehaf481_supplementary_data.docx]

**Supplementary Files**

**I. Trial organization**

**Principal Investigator:** Jing Chen (Department of Cardiology, Renmin Hospital of Wuhan University, Wuhan, China).

**Co-Principal Investigator:** Hong Jiang (Department of Cardiology, Renmin Hospital of Wuhan University, Wuhan, China).

**Steering Committee:** Jing Chen (Chair); Hong Jiang (Chair); Jian Yang (Department of Cardiology, The First College of Clinical Medical Science, China); Jun Wan (Department of Cardiology, Renmin Hospital of Wuhan University, Wuhan, China); Bo Yang (Department of Cardiology, Renmin Hospital of Wuhan University, Wuhan, China); Changwu Xu (Department of Cardiology, Renmin Hospital of Wuhan University, Wuhan, China)

**Project Manager:** Changwu Xu (Department of Cardiology, Renmin Hospital of Wuhan University, Wuhan, China).

**Clinical Events Committee:** Hairong Wang (Department of Cardiology, Zhongnan Hospital of Wuhan University, China); Yumiao Wei (Department of Cardiology, Union Hospital, Tongji Medical College, Huazhong University of Science and Technology, China); Yushi Wang (Department of Cardiology, The first hospital of Jilin University, China); Dan Tian (Department of Cardiology, Renmin Hospital of Wuhan University, Wuhan, China);

**Data and Safety Monitoring Board:** Shanshan Guo (Department of Cardiology, Renmin Hospital of Wuhan University, Wuhan, China); Liyue Wang (Wuchang Hospital Affiliated to Wuhan University of Science and Technology, China); Haoyue Li (Department of Cardiology, Renmin Hospital of Wuhan University, Wuhan, China); Jing Zhang (Department of Cardiology, The First College of Clinical Medical Science, China)

**Contract Research Organization:** KINGYEE (BEIJING) CO., LTD

**Core Laboratory for Coronary Angiograms Analysis:** Department of Cardiology, Nanjing First Hospital, Nanjing Medical University, China

**Core Laboratory for ECG Analysis:** Department of Cardiology, Zhongnan Hospital of Wuhan University, China

**Data Analysis Center:** Department of Epidemiology and Biostatistics, School of Public Health, Tongji Medical College, Huazhong University of Science and Technology, China

**II. Participating centers and investigators**

|  | Medical Center | Approval time for NCPC | Site Investigator(s) | Patients Randomized |
| --- | --- | --- | --- | --- |
| 1 | Xianning Central Hospital, Xianning, China | November 2018 | Bin Li | 170 |
| 2 | Renmin Hospital of Wuhan University, Wuhan, China | October 2016 | Jing Chen | 148 |
| 3 | Suizhou Central Hospital, Suizhou, China | April 2021 | Wei Yao | 77 |
| 4 | Yichang Central People's Hospital, Yichang, China | October 2016 | Jian Yang/Hui Wu | 65 |
| 5 | Tongcheng People's Hospital, Xianning, China | October 2016 | Longcai Hu | 50 |
| 6 | Ezhou Central Hospital, Ezhou, China | October 2016 | Guang Xu | 47 |
| 7 | Xiantao First People's Hospital, Xiantao, China | October 2016 | Dongmei Zhu /Zhengzai Li | 45 |
| 8 | Xiangyang Central Hospital, Xiangyang, China | May 2018 | Xiaolin Wu | 42 |
| 9 | Songzi People's Hospital, Jingzhou, China | October 2016 | Chuang Xiao | 40 |
| 10 | People's Hospital of Jingshan, Jingmen, China | April 2023 | Bo Liu | 27 |
| 11 | Chibi General Hospital, Xianning, China | July 2019 | Xiuzhen Shen | 24 |
| 12 | Jiangxia First People's Hospital, Wuhan, China | May 2021 | Zhaowu Deng | 21 |
| 13 | Anlu People's Hospital, Xiaogan, China | August 2022 | Chaogui Zhuo | 21 |
| 14 | Caidian People's Hospital, Wuhan, China | July 2019 | Huajun Su | 20 |
| 15 | Jingzhou Central Hospital, Jingzhou, China | September 2018 | Keping Yang | 17 |
| 16 | Shiyan People's Hospital, Shiyan, China | July 2017 | Youen Zhang | 16 |
| 17 | Wuhan Puren Hospital, Wuhan, China | April 2017 | Meichun Zhang | 15 |
| 18 | Hubei Zhongshan Hospital, Wuhan, China | December 2018 | Chang Li | 15 |
| 19 | CR & WISCO General Hospital, Wuhan, China | April 2017 | Xuexiang Lv | 14 |
| 20 | Wuhan Fifth Hospital, Wuhan, China | April 2017 | Lifeng Hong/Fan Guo | 14 |
| 21 | General Hospital of Yangtze River shipping, Wuhan, China | September 2017 | Xingan Wu | 12 |
| 22 | Guoyao Dongfeng General Hospital, Shiyan, China | July 2016 | Hao Xu | 12 |
| 23 | Xiangyang Hospital of Traditional Chinese Medicine, Xiangyang, China | October 2023 | Mingjian Li | 10 |
| 24 | Wuhan No.1 Hospital, Wuhan, China | August 2017 | Liqun He | 10 |
| 25 | Xishui People's Hospital, Huanggang, China | August 2021 | Wenjun Wu | 9 |
| 26 | The Central Hospital of Enshi Tujia And Miao Autonomous Prefecture, Enshi, China | December 2018 | Yuhua Lei | 9 |
| 27 | Wuhan No.3 Hospital, Wuhan, China | July 2017 | Dongsheng Li | 8 |
| 28 | Huanggang Central Hospital, Huanggang, China | November 2020 | Huoping Li/Shaoze Chen | 7 |
| 29 | Jiangling People's Hospital, Jingzhou, China | April 2021 | Hong Bao | 7 |
| 30 | Laohekou First Hospital, Xiangyang, China | November 2019 | Xuguang Xiong | 6 |
| 31 | Wuhan No.6 Hospital, Wuhan, China | November 2019 | Bo Liu | 5 |
| 32 | Wuhan Hanyang Hospital, Wuhan, China | December 2017 | Guokang Yang | 5 |
| 33 | Wuxue First People's Hospital, Huanggang, China | August 2022 | Jinke Chen/Xiao Lan | 4 |
| 34 | Xianning First People's Hospital, Xianning, China | June 2024 | Qibin Zheng | 4 |
| 35 | Tianmen First People's Hospital, Tianmen, China | March 2018 | Guanglong Yang | 2 |
| 36 | Wuhan Hospital of Traditional Chinese Medicine, Wuhan, China | November 2019 | Mingxi Zhang | 1 |

**III. Study Protocol**

**Early Administration of Heparin at First Medical Contact Versus in the Cath lab for STEMI Patients Undergoing Primary Percutaneous Coronary Intervention (HELP-PCI): A Multicenter, Randomized Trial**

**Study Protocol**

Clinical Trial Registration: NCT05329155

**1. Background**

Primary percutaneous coronary intervention (PPCI) is the established standard of care for patients presenting with acute ST-segment elevation myocardial infarction (STEMI) within 12 hours of symptom onset, which can effectively improve vessel patency, preserve cardiac function, and improve survival rates ^[1-2]^. Antithrombotic therapy is an important part of perioperative therapy for PPCI in STEMI patients. At present, unfractionated heparin (UFH) is the predominant anticoagulant for PPCI, typically administered in the catheterization laboratory with an initial dose of 70-100U/kg, titrated to maintain an activated clotting time (ACT) between 250-300 seconds ^[1]^. However, there is often a time delay from the first medical contact (FMC) to wire crossing in the catheterization laboratory (Cath Lab), ranging from several minutes to even hours in routine clinical practice, and early UFH treatment is widely thought to restore coronary blood flow earlier, thereby improving cardiac function.

Several retrospective studies suggested that early UFH treatment could achieve early restoration of blood flow in infarct-related artery (IRA) and even improve cardiac function in STEMI patients treated with PPCI ^[3-12]^, while other retrospective studies found negative results ^[13-14]^. Notably, large-scale, randomized controlled trials evaluating UFH pretreatment in STEMI patients before PPCI are lacking. Therefore, we hypothesized that a 100 U/kg loading dose of UFH administered at the FMC would result in earlier coronary blood flow restoration and improved clinical outcomes compared to UFH administration in the Cath Lab.

**2. Study Objective**

This investigator-initiated, prospective, multicenter, randomized (1:1), open-label, study (HELP-PCI) aims to evaluate the clinical efficacy and safety of pretreatment with UFH at FMC vs. in the Cath Lab for STEMI patients undergoing PPCI at 30 days, 6, and 12 months.

**3. Basic information on UFH**

UFH, initially isolated from the liver, is a highly sulfated, viscous polysaccharide composed of repeating disaccharide units of alternating glucosamine and uronic acid residues, including L-iduronic acid, D-glucuronic acid, and N-acetylglucosamine. Characterized by an average molecular weight of 15 kDa and strong acidity, UFH is also present in tissues such as the lungs, vascular endothelium, and intestinal mucosa, where it functions as a natural anticoagulant. At present, UFH is mainly extracted from bovine lung or porcine intestinal mucosa for clinical use. As a potent anticoagulant, UFH exerts its effects both *in vivo* and *in vitro* through its polymeric structure. Clinically, it is indicated for the treatment and prevention of thromboembolic disorders, including myocardial infarction, and is utilized in cardiovascular surgery, cardiac catheterization, extracorporeal circulation, and hemodialysis procedures.

Currently, UFH is the preferred anticoagulant for interventional treatment of coronary artery disease. In patients undergoing emergency PCI, UFH provides effective, safe, and cost-effective anticoagulation. Intraoperative ACT monitoring is essential to maintain values within the therapeutic range of 250-300 seconds. In cases of UFH overdose and subsequent bleeding risk, protamine sulfate can be administered for neutralization, with a recommended ratio of 1 mg protamine sulfate per 100 U UFH. The UFH formulation and dosage utilized in the HELP-PCI trial was 12,500 U/2 mL. Manufacturers of the UFH used in the trial included Chengdu Haitong Pharmaceutical Co., Ltd., Jiangsu Wanbang Biochemical Pharmaceutical Co., Ltd., Tianjin Biochemical Pharmaceutical Co., Ltd., Huabei Pharmaceutical Group Co., Ltd., and Hebei Kaiwei Pharmaceutical Co., Ltd.

**4. STUDY DESIGN**

**4.1 Overall study design**

A total of 944 STEMI patients undergoing PPCI within 12 hours of symptom onset and with an anticipated time from FMC to balloon inflation of ≤ 120 min will be randomized. Most participating hospitals are certified National Chest Pain Centers (NCPC) in China, as approved by the Chinese Cardiovascular Association. Eligibility will be determined based on predefined inclusion and exclusion criteria. Eligible patients will be randomly assigned to either the experimental or control group. The experimental group will receive an intravenous loading dose of 100 U/kg UFH at FMC within 10 minutes of STEMI diagnosis, while the control group will receive 100 U/kg UFH via the arterial sheath in the Cath Lab. All subjects will undergo clinical follow-up before discharge and at scheduled post-randomization visits.

**
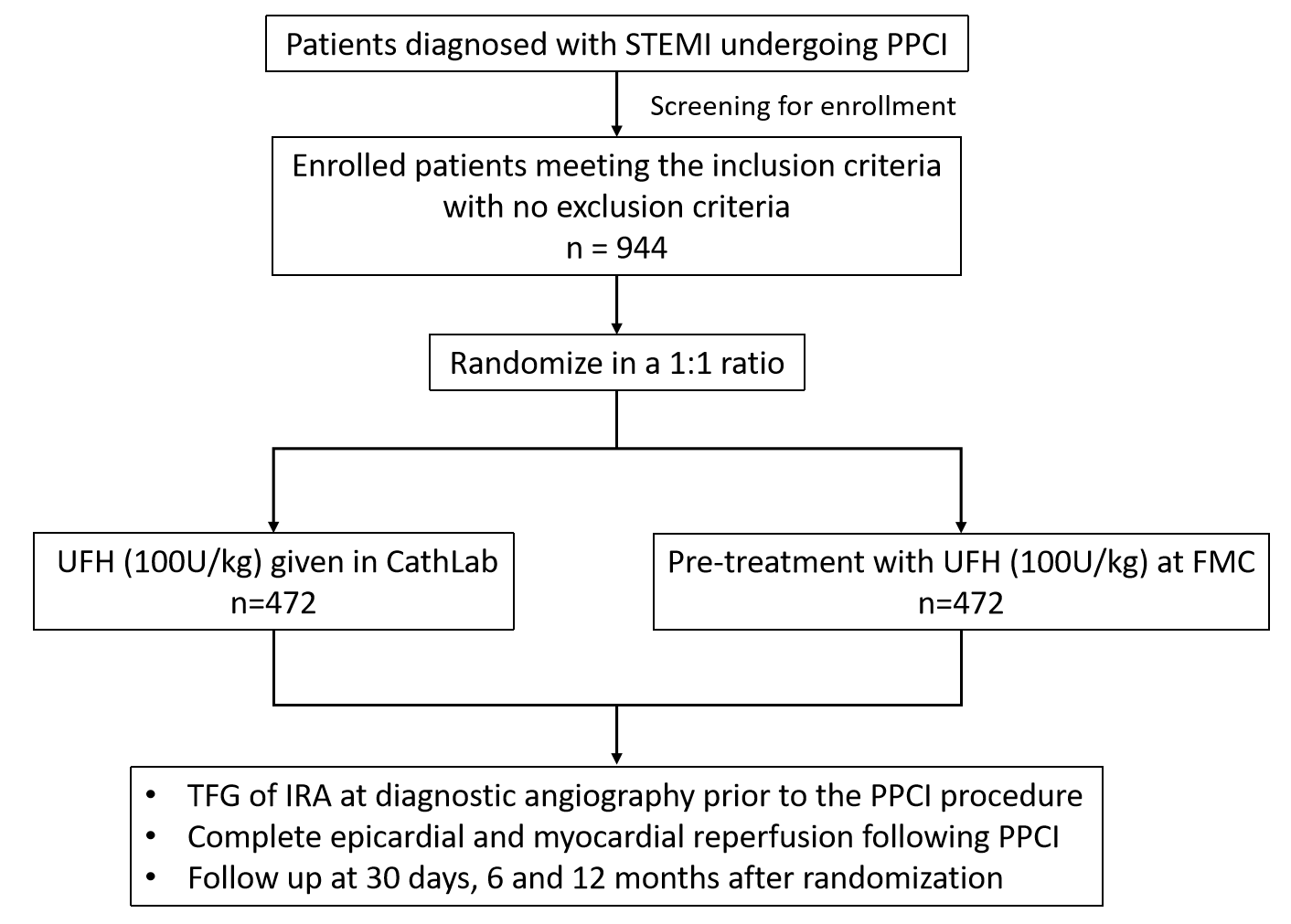
**

**Figure 1. HELP-PCI Trial Design**

**4.2 Treatment**

All STEMI patients meeting the inclusion and exclusion criteria will be randomized in a 1:1 ratio to either the experimental group or the control group.

The experimental group will receive a loading dose of dual antiplatelet therapy (DAPT), comprising 300 mg aspirin and either 180 mg ticagrelor or 300-600 mg clopidogrel, in conjunction with an intravenous loading dose of UFH at 100 U/kg, administered at FMC following diagnostic electrocardiogram (ECG). The pre-treatment UFH will be administered intravenously within 10 min of randomization and completed prior to transfer to the Cath Lab.

In the control group, patients will receive a loading dose of DAPT as described before at FMC, without prior administration of UFH. Following arterial sheath placement in the Cath Lab, 100 U/kg UFH will be administered via the arterial sheath, preceding PPCI. The precise intraprocedural UFH dosage, however, will be subject to the interventional cardiologist’s clinical evaluation and decision.

The monitoring of ACT is recommended for all patients undergoing PPCI. Supplemental UFH is indicated during PPCI when the ACT is below 225 seconds, or 1000 U UFH will be administered hourly during patient transport ^[5]^ or the PPCI procedure, based on the physician's clinical judgment, following the initial UFH administration.

Pretreatment with low molecular weight heparin (LMWH), bivalirudin, fondaparinux sodium, glycoprotein IIb/IIIa inhibitor (GPI), or other antithrombotic medications will be prohibited in all patients prior to PPCI. Provisional use of GPI will be permitted during or after PPCI at the investigator's discretion. GPI will be recommended in the presence of the following conditions: slow or no-reflow phenomenon (Thrombolysis in Myocardial Infarction flow grad 0-2), high thrombotic burden, new or suspected thrombus formation, persistent residual stenosis, distal embolization, and suboptimal stent implantation.

All patients will receive guideline-recommended prescriptions for anti-myocardial remodeling, lipid-lowering therapy, post-interventional antithrombotic therapy, and other indicated medications ^[1]^.

PPCI is defined as coronary artery intervention, encompassing thrombectomy, balloon angioplasty, or stent implantation, performed on the IRA without prior thrombolytic therapy. Radial artery access is the preferred approach; however, in specific clinical scenarios, alternative vascular access sites, such as the femoral, brachial, or ulnar arteries, may be utilized as deemed appropriate by the interventional cardiologist. In patients presenting with multivessel disease, the interventional cardiologist will determine the feasibility of complete revascularization during the index PPCI procedure. If immediate multivessel PCI is not feasible, and the patient's clinical condition is stable, staged PCI is recommended to be performed after 30 days post-randomization.

Time points will be recorded by each participating center before and during PPCI on the designated pages of the case report form (CRF). Weekly unified clock calibration will be performed at each center. In accordance with NCPC requirements, clock calibration will be conducted in the emergency department, Cath Lab, cardiology intensive care unit, outpatient department, ECG department, and on digital subtraction angiography (DSA) machines, among other relevant locations. 12- or 18-lead ECG will be obtained upon admission and 90 minutes post-PPCI. Subsequent standard intensive care unit management includes close monitoring of vital signs and general patient condition, encompassing consciousness, pulse, heart rate, rhythm, respiratory rate, blood pressure, body temperature, blood oxygen saturation, and 24-hour fluid balance. Dynamic recording of ECG, cardiac ultrasound, myocardial injury biomarkers, B-type natriuretic peptide (BNP) or N-terminal pro-B-type natriuretic peptide (NT-proBNP), D-dimer, complete blood count, coagulation parameters, liver and renal function tests, blood glucose, lipid profile, and electrolytes will be performed.

Telephone or outpatient follow-up will be conducted at 30 days, 6 months, and 12 months post-randomization. In the event of an adverse event, participants will be instructed to contact their physicians immediately.

**4.3 Randomization**

Following the provision of informed consent, patients meeting all inclusion criteria and none of the exclusion criteria will be assigned to either the experimental or control group via a centralized randomization system. Randomization will be performed using a pre-set program developed by the Clinical Research Center of the Cardiovascular Research Institute at Renmin Hospital of Wuhan University. A randomized block design will be implemented by statisticians who did not participate in the study. Researchers will utilize the Interactive Web Response System (IWRS) to contact the randomization center and input information as instructed. All eligible patients will be randomly assigned to either the experimental or control group through the IWRS. The IWRS will assign a unique randomization number (subject ID) to each patient and simultaneously track enrollments from all participating sites.

**4.4 Blinding**

This study employs a single-blind design. While investigators and participants are aware of the treatment allocation, the independent Clinical Events Committee (CEC) and the independent core laboratory will remain blinded to the group assignments.

**4.5 Schedule of data collection**

Table 1. Schedule of data collection

| Study procedures | V1 | V2 | V3 | V4 | V5 |
| --- | --- | --- | --- | --- | --- |
|  | PPCI | Discharge | 30±7d | 180±30d | 365±30d |
| Inclusion/exclusion criteria | X |  |  |  |  |
| Patient Informed Consent | X |  |  |  |  |
| Randomization | X |  |  |  |  |
| Medical History/ Demographics | X |  |  |  |  |
| Brief physical examination ^1^ | X | X |  |  |  |
| Vital Signs ^2^ | X | X |  |  |  |
| Routine laboratory ^3^ | X |  |  |  |  |
| Cardiac Markers | X |  |  |  |  |
| 12/18-Leads ECG ^4^ | X |  |  |  |  |
| Angiography and PPCI ^5^ | X |  |  |  |  |
| Time recorder | X |  |  |  |  |
| Clinical Assessment | X | X | X | X | X |
| Concomitant Medications | X | X | X | X | X |
| Echocardiography ^6^ | X |  |  |  |  |
| Adverse events | X | X | X | X | X |

1. Killip classification will be assessed as follows:

Killip class I: Absence of rales over the lung fields and absence of an S3 gallop;

Killip class II: Rales present over 50% or less of the lung fields or the presence of an S3 gallop;

Killip class III: Rales present over more than 50% of the lung fields;

Killip class IV: Cardiogenic shock or signs include hypotension (systolic pressure of 90 mmHg or less) and evidence of peripheral vasoconstriction such as oliguria, cyanosis and diaphoresis.

2. Vital signs: Heart rate, systolic blood pressure (BP), and diastolic BP will be assessed on admission.

3. A complete blood count will be obtained upon admission, with subsequent testing during the in-hospital period to be determined at the discretion of the attending physician(s).

4. ECGs will be obtained at FMC and 90 minutes after PPCI.

5. TIMI flow grade (TFG) and TIMI myocardial perfusion grade (TMPG) will be evaluated as the study endpoints. Thrombosis burden will be classified using TIMI thrombus grading. Multivessel coronary artery disease is defined as the presence of angiographic stenosis of at least 70% in at least one non-culprit coronary artery with a diameter of at least 2.25 but no more than 5.75 mm or left main stem stenosis of more than 50%, as estimated by visual assessment ^[15]^. Complete revascularization is defined as PPCI of the IRA along with simultaneous or routine staged PCI of all suitable non-infarct-related arteries (N-IRA) during the index admission.

5. The first echocardiography will be scheduled within 24 hours post-PPCI.

**5. STUDY POPULATION**

**5.1 Inclusion criteria (all must be present)**

1) Age 18 to 80 years

2) Presentation of STEMI within 12 hours of symptom onset. STEMI is defined as: ST-segment elevation ≥ 1 mm in ≥ 2 contiguous leads (≥2 mm elevation in leads V_2_-V_3_), or New left bundle branch block (LBBB) or right bundle branch block (RBBB) with ST-segment elevation or Q waves in leads V_1_-V_3_.

3) Planned PPCI with an anticipated time from FMC to balloon inflation of ≤ 120 min and without planned thrombolytic therapy.

**5.2 Exclusion criteria (all must be absent):**

1. Mechanical complications of MI (e.g., ventricular septal rupture or severe acute mitral regurgitation, etc.)
2. Cardiopulmonary resuscitation before randomization
3. History of coronary artery bypass grafting (CABG)
4. Active bleeding
5. Life expectancy < 1 year
6. History of heparin-induced thrombocytopenia
7. Current use of anticoagulation medications
8. Pregnancy or lactation
9. Refusal to provide signed informed consent

**5.3 Exclusion of Patients**

**5.3.1 Withdrawal Criteria and Procedures**

Patients will be withdrawn from the trial prior to completion if any of the following criteria are met: 1) Post-recruitment discovery of inconsistency with the inclusion or exclusion criteria; 2) Voluntary withdrawal of consent by the subject; 3) Inability of the subject to undergo emergency angiography due to critical clinical conditions or other medical reasons; 4) Determination by the investigators that continued participation in the trial is no longer in the best interest of the subject;

Unnecessary patient withdrawal will be avoided. For all withdrawals, the reason and time will be recorded in the CRF or source documents. Adverse events leading to withdrawal, if judged related to the experimental method, must be documented in the CRFs and reported to the investigators. Subjects withdrawing due to adverse events require follow-up until a definitive diagnosis and treatment.

**5.3.2 Criteria for treatment discontinuation**

Study medication discontinuation will be considered for patient refusal, intolerable side effects, or investigator-determined termination due to severe complications or inappropriate situations necessitating cessation of treatment. All reasons for discontinuation will be clearly documented within the CRFs.

**6. STUDY CONDUCTION**

**6.1** **Screening and Enrollment Period**

Following approval from the local ethics committee (EC), researchers will screen potential participants according to the predefined inclusion and exclusion criteria, and eligible subjects will be required to provide signed informed consent via an ICF prior to enrollment; individuals who do not meet the eligibility criteria or who are not ultimately randomized will be documented in a screening log.

The STEMI diagnosis will be based on the following four criteria in this trial:

1. Ischemic chest pain or equivalent symptoms persisting for at least 30 minutes;
2. New ischemic ECG alterations meeting at least one of these criteria-ST-segment elevation ≥ 1 mm in ≥ 2 contiguous leads (≥ 2 mm elevation in leads V_2_-V_3_), new LBBB, or RBBB with ST-segment elevation or Q waves in leads V_1_-V_3_;
3. Symptom onset within 12 hours of symptom onset.

**6.2** **Informed consent**

Following informed consent, obtained through a signed ICF, all trial procedures will be executed. Researchers or designated personnel trained in this protocol will comprehensively explain the trial's nature, scope, potential risks, and benefits to prospective participants, addressing any inquiries. Upon agreement to participate, the ICF must be signed and personally dated by the subject or a legally authorized representative.

Given the emergency nature of this study, where patients present in critical condition or the time for informed consent review prior to randomization is severely limited, meticulous documentation of all screened patients, including the reasons for screening failures and non-randomization, is essential throughout the enrollment period.

Prior to randomization, a comprehensive ICF with the patient’s signature or that of their legally authorized representative is required. However, recognizing the emergent nature of this trial, and in alignment with recommendations from the IABP-SHOCK II and ALTNTC trials ^[16-17]^, the EC of Renmin Hospital of Wuhan University and local ethics committees have approved the following consent procedures: 1) If the patient is incapable of signing, two independent medical personnel shall document the patient's or representative's wishes via written or audio record before randomization; 2) In time-critical situations or when the patient's condition precludes signing the full ICF, a simplified ICF may be signed before randomization; 3) If only the simplified ICF is signed, or if the patient's condition initially prevented signing, the full ICF must be obtained as soon as clinically feasible before discharge. Should the patient or representative subsequently refuse to complete the full consent process, the patient will be withdrawn from the trial following thorough discussion, and no further data will be collected or used.

**6.3 Treatment plan**

**6.3.1 PPCI**

PPCI is the preferred reperfusion strategy for acute STEMI patients when the anticipated time from FMC to balloon inflation is ≤ 120 minutes, aiming for rapid restoration of blood flow and cardiac function preservation; in such cases, thrombolytic therapy is contraindicated. PPCI is defined as intervention of the IRA without antecedent thrombolytic therapy, encompassing stent implantation, balloon angioplasty, or thrombectomy. For patients with multivessel disease, if immediate multivessel PCI is not feasible and the patient's clinical condition permits, staged PCI is recommended to be performed after 30 days post-randomization.

**Definition of FMC**

In the context of STEMI reperfusion therapy, FMC denotes the precise time at which a patient's ECG is definitively recognized and diagnosed as STEMI by medical personnel, whether in the emergency department or pre-hospital ambulance setting. The interval between this initial STEMI diagnosis and the patient's arrival at the Cath Lab constitutes the FMC period, which in this study's experimental group, represents the time of UFH administration.

**6.3.2 UFH administration**

1. **UFH pretreatment at FMC (experimental group)**

Following diagnostic ECG and randomization, the experimental group will receive a loading dose of DAPT, comprising 300 mg aspirin and either 180 mg ticagrelor or 300-600 mg clopidogrel, concurrently with an intravenous loading dose of UFH at 100 U/kg at FMC. UFH pretreatment must be initiated within 10 minutes of randomization and completed prior to patient transfer to the Cath Lab.

1. **UFH treatment in the Cath Lab (control group)**

In the control group, patients will receive a loading dose of DAPT, comprising 300 mg aspirin and either 180 mg ticagrelor or 300-600 mg clopidogrel, at FMC, without initial UFH administration. Subsequently, following arterial sheath placement in the Cath lab, 100 U/kg UFH will be administered through the arterial sheath prior to PPCI, with the final acute UFH dosage determined at the discretion of the interventional cardiologist.

In both study groups, intraoperative ACT monitoring during PPCI will be strongly recommended for all patients. Supplemental UFH administration will be permitted during PPCI if the ACT falls below 225 seconds, or, based on clinical judgment, 1000 U UFH will be administered hourly during patient transport and the PPCI procedure following the initial UFH dose.

**6.3.3 Concomitant medications**

**Dual Antiplatelet Therapy**

In accordance with NCPC recommendations and current guidelines ^[1]^, antiplatelet pretreatment will be administered within 20 minutes of STEMI diagnosis. Specifically, a 300 mg loading dose of chewable aspirin is recommended prior to PPCI. P2Y12 inhibitor pretreatment will be encouraged, utilizing a 180 mg loading dose of ticagrelor or 300-600 mg of clopidogrel, with the selection of P2Y12 inhibitor determined by the investigators. Post-PPCI, DAPT will be recommended for 12 months, consisting of a 100 mg daily maintenance dose of aspirin and either 75 mg daily clopidogrel or 90 mg ticagrelor twice daily.

**Other antithrombotic drugs**

Prior to PPCI, pretreatment with LMWH, bivalirudin, fondaparinux sodium, GPIs, or other antithrombotic medications was prohibited. Post-PPCI anticoagulant use, including LMWH and UFH, will be left to the investigator's discretion, guided by European Society of Cardiology (ESC) guidelines ^[1]^.

Provisional GPI will be permitted during or after PPCI at the investigator’s discretion, with recommendations favoring their use in cases of slow or no-reflow (TIMI 0-2), high thrombotic burden, new or suspected thrombosis, persistent residual stenosis, distal embolization, suboptimal stent implantation, or other clinically relevant scenarios; if deemed necessary, intravenous tirofiban, the domestically available GPI, will be initiated with a 25 μg/kg bolus infusion over 3-5 minutes, followed by a maintenance infusion of 0.15 μg/kg/min for up to 18 hours, with the maintenance dose reduced to 0.075 μg/kg/min for patients with an estimated glomerular filtration rate (eGFR) < 60 mL/min ^[1]^.

**6.4 Follow-up**

Patients enrolled in this study will undergo follow-up assessments at 30 ± 7 days, 180 ± 30 days, and 365 ± 30 days post-randomization.

**7. Study endpoints and Definitions**

**7.1 Endpoint**

**7.1.1 Primary endpoint**

TFG-3 of IRA at diagnostic angiography, performed prior to the PPCI procedure, will be evaluated as a key angiographic outcome.

**7.1.2 Secondary endpoint**

1. The incidence of major adverse cardiac and cerebrovascular events (MACCE), a composite endpoint encompassing all-cause death, cardiac death, hospitalization for heart failure, myocardial infarction, stent thrombosis, unplanned revascularization, and stroke, as well as the incidence of each individual component of MACCE, at 1-year post-randomization;
2. Complete epicardial and myocardial reperfusion following PPCI, defined as the

simultaneous attainment of TIMI flow grade 3, TMPG grade 3, and ST-segment resolution ≥ 70% at 90 minutes post-PPCI.

**7.1.3 Safety endpoint**

The primary safety endpoint for this study is the incidence of Bleeding Academic Research Consortium (BARC) type ≥ 2 bleeding events within 30 days following randomization, thereby assessing the risk of clinically significant bleeding associated with the experimental intervention.

**7.2 Assessment of ECG and Angiography**

**7.2.1 Angiography**

Pre- and post- PPCI coronary angiography will be conducted in accordance with core laboratory guidelines and local clinical practice, with epicardial artery flow assessed using TFG and myocardial tissue-level perfusion evaluated via TMPG. To ensure high-quality imaging, angiography will be performed with a manual injection of 6-10 mL of contrast agent over a sufficient duration, and cine filming will extend beyond three cardiac cycles in the washout phase, requiring a minimum of 10 seconds from contrast injection. Filming at 25 or 30 frames per second with at least two projections is preferred; from multiple orthogonal projections, the single view best visualizing the distal coronary artery will be selected, typically right anterior oblique (RAO) with cranial angulation for the left anterior descending artery (LAD), RAO caudal or left lateral for the left circumflex artery (LCX), and either left anterior oblique or RAO for the right coronary artery (RCA) distribution ^[18]^.

**Centralized assessment of angiographic parameters**

Imaging data acquired pre- and post-PPCI will undergo centralized assessment at an independent laboratory (Department of Cardiology, Nanjing First Hospital, Nanjing Medical University, China) by experienced readers blinded to treatment assignment and clinical outcomes. Specifically, the following parameters will be evaluated: TFG of the IRA pre- and post-PPCI angiography, TMPG pre- and post-PPCI angiography, and thrombus burden pre- and post-PPCI angiography.

**TFG grading**

The TFG assessment, utilized in coronary angiography, is categorized as follows:

TIMI 0 flow (no perfusion) refers to the absence of any antegrade flow beyond a coronary occlusion;

TIMI 1 flow (penetration without perfusion) is faint antegrade coronary flow beyond the occlusion, with incomplete filling of the distal coronary bed;

TIMI 2 flow (partial reperfusion) is delayed or sluggish antegrade flow with complete filling of the distal territory;

TIMI 3 flow (complete perfusion) is normal antegrade flow resulting in complete distal coronary bed filling.

**TMPG grading**

TMPG is a standardized angiographic assessment tool used to evaluate the efficacy of reperfusion therapy in restoring myocardial blood flow. This evaluation, conducted via coronary angiography, categorizes flow within the IRA and its distal vascular bed into four distinct grades, thereby providing a quantitative measure of myocardial perfusion. Grading will be conducted as follows:

TMPG 0: No Perfusion

- Complete occlusion of the IRA with no antegrade flow beyond the occlusion.

TMPG 1: Penetration Without Perfusion

- Contrast material passes beyond the area of obstruction but fails to opacify the entire coronary bed distal to the obstruction.

TMPG 2: Partial Perfusion

- Contrast material passes through the obstruction and opacifies the coronary bed distal to the obstruction, but the rate of entry or clearance of contrast material is slower than in non-IRA.

TMPG 3: Complete Perfusion

- Antegrade flow into the bed distal to the obstruction occurs as promptly as into the non-IRA, and clearance of contrast material is as rapid as in non-IRA.

**TIMI Thrombus Burden Grading**

The TIMI Thrombus Burden grading system provides a standardized angiographic assessment of intracoronary thrombus size and extent in patients with STEMI, classifying thrombi into six grades:

Grade 0 (No thrombus): No angiographic characteristics of thrombus present.

Grade 1 (Possible thrombus): Angiographic features suggestive of thrombus, such as reduced contrast density, haziness, or irregular lesion contour, but without clear thrombus visualization.

Grade 2 (Small thrombus): Definite thrombus with a greatest dimension≤1/2 the vessel diameter.

Grade 3 (Moderate thrombus): Definite thrombus with a greatest dimension > 1/2 but < 2 vessel diameters.

Grade 4 (Large thrombus): Definite thrombus with a greatest dimension≥2 vessel diameters.

Grade 5 (Very large thrombus): Complete occlusion of the vessel due to thrombus, with no antegrade flow (TIMI flow grade 0).

**7.2.2 ECG**

12- or 18-lead ECG will be performed according to local clinical practice upon admission and 90 minutes after PCI. Standard ECG recordings, including a lead II rhythm strip, will be obtained with the patient in a supine position, with particular attention to ST-segment measurement. All original ECG recordings will be retained and stored within the CRFs as source documentation.

To ensure an objective assessment of ST-segment elevation, an independent central review will be performed blindly by an independent core laboratory (Department of Cardiology, Zhongnan Hospital of Wuhan University, China), utilizing three readings and calculating the mean. The sum of ST-segment elevation, measured 20 ms after the J point and compared to the baseline ECG, will be used to calculate ST-segment elevation resolution, defined as the difference between the initial and post-PPCI sum of ST-segment elevations divided by the initial sum, expressed as a percentage. Complete resolution will be defined as a resolution of ≥70%, with incomplete resolution categorized as partial (30-70%) or absent (<30%) resolution, as per established criteria ^[18]^. Only patients with both pre- and post-PPCI ST-segment elevation measurements will be included in this calculation.

**7.3 Clinical Outcomes**

**7.3.1 All Cause Death**

Defined as any death, including both cardiac and non-cardiac causes.

**7.3.2 Cardiac death**

All deaths occurring during the study period will be considered cardiogenic, encompassing those resulting from acute myocardial infarction, sudden cardiac death, low output heart failure, fatal arrhythmia, unobserved or unexpected death, and out-of-hospital cardiac arrest with successful resuscitation but subsequent in-hospital mortality. This includes deaths in subjects with severe non-cardiac disease. In cases where the cardiovascular or non-cardiovascular nature of death cannot be definitively ascertained, the death will be classified as cardiogenic.

**7.3.3 Re-admission for heart failure**

Readmission for heart failure will be defined as any condition meeting either the composite criteria of (1+2+3) or criterion 4. Specifically, the composite criteria require: 1) at least 24 hours of treatment within a hospital ward or emergency department; 2) the presence of symptoms or signs of heart failure; and 3) the administration of intravenous medications (e.g., diuretics, vasoactive agents) or invasive therapies (e.g., intra-aortic balloon pump, mechanical ventilation, ultrafiltration) for heart failure management. Alternatively, readmission for heart failure will be defined by criterion 4, which encompasses either death within 24 hours of emergency department arrival due to heart failure despite resuscitative efforts, or hospitalization for heart transplantation.

**7.3.4 Myocardial infarction (re-infarction)**

In this study, 'myocardial infarction (MI) ' and 're-infarction' are defined consistently, with re-infarction representing a subsequent acute myocardial infarction occurring during the follow-up period. Both myocardial infarction and re-infarction are defined according to the criteria outlined in the fourth Universal Definition of Myocardial Infarction.

**Definition of Myocardial Infarction:**

- Acute myocardial infarction is diagnosed when there is evidence of acute myocardial injury, indicated by a rise and/or fall of cardiac troponin values with at least one value exceeding the 99th percentile upper reference limit (URL), accompanied by clinical evidence of acute myocardial ischemia as demonstrated by one or more of the following:

- Symptoms of myocardial ischemia.

- New ischemic ECG changes.

- Development of pathological Q waves.

- Imaging evidence of new loss of viable myocardium or new regional wall motion abnormality in a pattern consistent with ischemic etiology.

- Identification of a coronary thrombus by angiography or autopsy.

In patients presenting with clinical suspicion of re-infarction, immediate troponin (cTn) testing followed by a repeat measurement 3-6 hours later is recommended. If the baseline cTn is elevated but stable or decreasing, re-infarction is defined by a cTn increase of ≥50% over a prior decreasing value, in conjunction with either recurrent ischemic cardiac symptoms exceeding 20 minutes at rest or new ischemic ECG changes; conversely, if the baseline cTn is within the normal range, the diagnostic criteria for newly diagnosed acute MI should be applied, requiring at least one cTn value above the 99th percentile upper reference limit, accompanied by symptoms of myocardial ischemia and corroborative ECG or imaging findings.

**Types of Myocardial Infarction**

Type 1 MI: Spontaneous myocardial infarction related to atherosclerotic plaque rupture, ulceration, fissuring, erosion, or dissection with resulting intraluminal thrombus in one or more of the coronary arteries leading to decreased myocardial blood flow or distal platelet emboli with ensuing myocyte necrosis.

Type 2 MI: Myocardial infarction secondary to an ischemic imbalance due to either increased oxygen demand or decreased supply, e.g., coronary artery spasm, coronary embolism, anemia, arrhythmias, hypertension, or hypotension.

Type 3 MI: Cardiac death with symptoms suggestive of myocardial ischemia and presumed new ischemic ECG changes or new LBBB, but death occurring before blood samples could be obtained or before cardiac biomarker values could rise.

Type 4 MI: Myocardial infarction associated with PCI.

Type 5 MI: Myocardial infarction associated with CABG.

**7.3.5 Stent thrombosis**

In accordance with the Academic Research Consortium (ARC) definitions, only definite stent thrombosis (ST) will be considered a primary endpoint. ST will be defined, based on surgical reports, as thrombus obstruction within a 5mm segment proximal or distal to the stent or within the stent area, resulting in TIMI blood flow grade 0. Alternatively, ST may be diagnosed by the presence of thrombus within a 5mm segment proximal or distal to the stent or within the stent area, with TIMI blood flow grades 1, 2, or 3, provided at least one of the following criteria is met within 48 hours: 1) ischemic symptoms at rest, exemplified by typical chest pain lasting 20 minutes; 2) new ischemic ECG changes indicative of acute ischemia; or 3) dynamic alterations in cardiac biomarkers.

**7.3.6 Unplanned revascularization**

Unplanned revascularization is defined as the performance of either percutaneous coronary intervention (PCI) or CABG surgery within 24 hours of the recurrence of myocardial ischemia symptoms when such intervention was not initially planned as part of the index procedure or a scheduled staged procedure.

**7.3.7 Stroke**

Stroke, for the purposes of this study, is defined as the new onset of a focal neurological deficit of vascular origin, manifesting with signs or symptoms persisting for more than 24 hours (or ≤24 hours with medical or interventional resolution). This neurological dysfunction, whether focal or systemic, must be accompanied by at least one of the following: altered consciousness, unilateral limb hemiplegia or hemiparesis, sensory impairment, dysphasia or aphasia, hemianopia, amaurosis fugax, or other neurological manifestations consistent with stroke. While neuroimaging via computerized tomography (CT) or magnetic resonance imaging (MRI) is recommended, it is not mandatory for inclusion. Both ischemic and hemorrhagic stroke subtypes will be considered primary endpoints and will be differentiated during event adjudication.

**7.3.8 BARC Bleeding**

Bleeding events were determined based on BARC classification (1-5 types):

**Type 1:**

The bleeding is not severe and will not lead the patient to seek unplanned follow-up, hospitalization, or professional medical treatment.

**Type 2:**

Any clinically overt sign of hemorrhage that is actionable but does not meet the criteria for types 3, 4, or 5 bleeding. It must meet at least one of the following criteria:

1) requiring medical or percutaneous intervention guided by a health care professional, including (but not limited to) temporary/permanent cessation of a medication, coiling, compression, local injection;

2) leading to hospitalization or an increased level of care;

3) prompting evaluation defined as an unscheduled visit to a healthcare professional resulting in diagnostic testing (laboratory or imaging).

**Type 3:**

Clinical, laboratory, and/or imaging evidence of bleeding with specific healthcare provider responses, as listed below:

**3a**

1) Any significant bleeding that requires blood transfusion

2) Obvious bleeding and a decrease in hemoglobin (Hb) of 3-5g/dL (indicating a correlation between hemoglobin decrease and bleeding)

**3b**

1) Obvious bleeding and Hb decrease≥5 g/dL (indicating a correlation between hemoglobin decrease and bleeding)

2) Cardiac tamponade;

3) Bleeding requiring surgical intervention for control (excluding dental/nasal/skin/hemorrhoid);

4) Intravenous injection of vasoactive drugs is required

**3c**

1) Intracranial hemorrhage (excluding cerebral microbleeds or hemorrhagic transformation; including intraspinal bleeding)

2) Subtypes confirmed by autopsy, imaging, or lumbar puncture

3) Bleeding that damages vision

**Type 4: CABG-Related Bleeding**

1) Intracranial hemorrhage within 48 hours.

2) Reoperation following closure of sternotomy for bleeding control;

3) Transfusion of≥5 units of whole blood or packed red blood cells within a 48-hour period;

4) Chest tube drainage ≥ 2 L within 24 hours

**Type 5: Fatal Bleeding.**

**5a**

Probable fatal bleeding: There is no autopsy or imaging confirmation, but there is a clinical suspicion of potentially fatal bleeding.

**5b**

Definite fatal bleeding: Clear, autopsy or imaging confirmed fatal bleeding.

**8. Safety evaluation and solution protocol**

**8.1 Adverse events**

Adverse events (AEs) in this clinical trial encompass any unfavorable medical occurrences during the study period, irrespective of a causal relationship to the intervention, excluding planned surgeries for pre-existing conditions without exacerbation.

Simple laboratory abnormalities are generally not classified as AEs, with exceptions made for: (1) clinically significant laboratory deviations indicating disease progression relative to baseline; (2) laboratory abnormalities necessitating medical intervention; or (3) laboratory abnormalities requiring participant withdrawal from the trial.

**8.2 Serious adverse events**

Study-related adverse events meeting any of the following criteria are classified as serious adverse events (SAEs):

1. Death or a life-threatening adverse event;
2. Persistent or significant disability/incapacity;
3. Hospitalization or prolongation of existing hospitalization;
4. A congenital anomaly/birth defect;
5. A medically important event that, based upon appropriate medical judgment, may jeopardize the patient or subject and may require medical or surgical intervention to prevent one of the outcomes listed above.

All SAEs as defined above must be reported within 24 hours after becoming aware of the event. Investigators are required to record the date of each SAE in the original data. Investigators should further report SAEs to local regulatory authorities in compliance with the requirements of their institution's ethics committee.

**8.3 The correlation between AEs/SAEs and drugs**

The causality assessment of AEs/SAEs with study drugs will be classified into the following categories: definitely related, very likely related, possibly related, possibly unrelated, and definitely unrelated.

The determination of whether a medical intervention may have caused or contributed to an adverse event is the responsibility of the investigator, and the conclusion must be documented in the corresponding CRFs. Investigators should evaluate the temporal relationship, biological plausibility, association with the patient's underlying disease, and the presence of alternative explanations before assigning causality.

**8.4 Assessment of the severity of AEs**

AE severity will be graded as follows: 1) Mild: Does not interfere with normal daily activities. 2) Moderate: Interferes with normal daily activities. 3) Severe: Prevents normal daily activities.

SAEs will be collected, summarized, and reported to the investigators, research team leaders, and ethics committees.

**8.5 AEs monitoring and Treatment**

During the clinical trial, investigators will diligently monitor each participant for the occurrence of AEs and SAEs. All AEs, regardless of the investigator's assessment of causality with the investigational drug, must be observed, reported, and documented in the CRFs. Investigators are responsible for providing appropriate medical management for all AEs.

All subjects are required to report AEs as previously defined, encompassing any new events or experiences not present at baseline, as well as exacerbations of pre-existing conditions. All newly acquired information regarding AEs must be documented in the corresponding CRFs. Subjects who have provided informed consent but have not participated in the trial are exempt from AE reporting.

Stable, chronic, or pre-existing conditions that exhibit no worsening are not considered AEs and should not be recorded in the CRFs.

**9. Data management**

**9.1 Data Collection and Storage**

Subject data will be collected using a secure Electronic Data Capture (EDC) system. The principal investigator or authorized associate investigator is responsible for ensuring the accuracy and completeness of all recorded data, and must provide a signature on the corresponding CRF. All required information as specified in the study protocol must be entered into the CRF, and explanations must be provided for any missing data. Data must be collected and accurately entered into the EDC as soon as it is obtained. Errors discovered in the CRF will be corrected by the investigators or designated personnel, as appropriate. All patient withdrawals or loss to follow-up must be documented in the CRF. All data will be validated according to the data validation plan, and detailed explanations must be provided for any data anomalies. If missing data, out-of-range values, or inconsistent or illogical data are identified, queries will be generated and provided to the investigators for clarification.

**9.2 Data Monitoring**

An independent Data and Safety Monitoring Board (DSMB) will monitor the accumulating safety and efficacy data. The DSMB members will be distinct from the investigators and the Steering/Executive Committee. The monitors will verify ICFs, enrollment information, data accuracy, record modifications, AE documentation, SAE reporting, and other relevant aspects.

**10. STATISTICAL ANALYSIS**

**10.1 Sample Size Calculation**

The study hypothesis is that pre-treatment with a full dose of UFH at FMC is superior to UFH administration in the Cath Lab with respect to the primary endpoint, TFG-3 in the IRA at diagnostic angiography prior to the PPCI procedure. The estimated incidence of the primary endpoint was determined based on prior similar PPCI studies (Table 1). Assuming an incidence of 15.5% for TIMI flow grade 3 after UFH administration at FMC and 9.2% after UFH administration in the Cath Lab, and accounting for an approximate 10% withdrawal rate, a total of 944 patients (472 evaluable patients per group) are planned for enrollment and randomized in a 1:1 ratio. This sample size provides 80% power to detect a 6.3% absolute difference in the primary endpoint between UFH administration at FMC and UFH administration in the Cath Lab, with a two-sided type I error rate of 0.05.

| **Studies** | **Number of cases** | **Study type** | **Treatment** | **TCFs 3 before PPCI** | |
| --- | --- | --- | --- | --- | --- |
|  |  |  |  | **UFH given at FMC** | **UFH given in cath lab** |
| HEAP ^[13]^ | 584 | RCT | UFH pretreatment vs. no pretreatment before PPCI | 13% | 9% |
| HEAP Pilot ^[10]^ | 100 | Registry | UFH pretreatment vs. no pretreatment before PPCI | 31% | 9% |
| TASTE ^[4]^ | 7244 | RCT  (Post Hoc) | UFH pretreatment vs. no pretreatment before PPCI | 13.1% | 8.1% |
| Felix Zijlstra etc. ^[8]^ | 1702 | Registry | UFH pretreatment vs. no pretreatment before PPCI | 17% | 10% |
| Enrico Fabris etc. ^[9]^ | 537 | Registry | UFH pretreatment vs. no pretreatment before PPCI | 14.3% | 9.7% |
| CHUS ^[12]^ | 709 | Registry | UFH loading in a non-PCI capable hospital before transfer vs. UFH in the catheterization laboratory | 44.6%  (TCF 2-3) | 18.5%  (TCF 2-3) |
| SCAAR ^[3]^ | 41,631 | Registry | UFH pretreatment vs. no pretreatment before PPCI | 62%  (TCF 0) | 71%  (TCF 0) |
| Victorian Cardiac Outcomes Registry ^[5]^ | 4720 | Registry | UFH pretreatment vs. no pretreatment before PPCI | 33.7%  (TCF 2-3) | 24.2%  (TCF 2-3) |
| Ninewells Hospital ^[14]^ | 1000 | Registry | UFH pretreatment vs. no pretreatment before PPCI | 25.4%  (TCF 2-3) | 24.2%  (TCF 2-3) |

**10.2 Analysis Data Set**

Full analysis set (FAS): The Full Analysis Set will adhere to the intention-to-treat principle, encompassing all randomized patients assigned to either intravenous UFH treatment at FMC (experimental group) or UFH treatment in the Cath Lab (control group), who have undergone emergent angiography assessment for the primary endpoint. The FAS will be utilized for baseline characteristic and primary endpoint analyses.

Per-protocol set (PPS): The Per-Protocol Set will consist of patients who met all inclusion and exclusion criteria and completed the treatment protocol without major protocol violations or premature discontinuation. The primary endpoint analysis will be repeated in the PPS to support the primary results obtained from the FAS.

Safety set (SS): The Safety Set will include all randomized patients who received at least one dose of the study drug and have documented safety data. Safety analyses will be performed using the SS.

**10.3 Statistical methods**

**10.3.1 General analysis**

Statistical analyses will be conducted by the Department of Epidemiology and Biostatistics, School of Public Health, Tongji Medical College, Huazhong University of Science and Technology, using R software (version 4.2.2; R Core Team) and Statistical Package for the Social Sciences software (version 26.0; IBM Crop). All statistical tests will be two-sided, and a *p*-value of < 0.05 will be considered statistically significant for all comparisons, unless otherwise specified. Continuous variables will be summarized as mean ± standard deviation (SD) or median with interquartile range (IQR), including the number of observations and missing values. Categorical variables will be reported as counts and percentages, including the number of observations and missing values. Baseline characteristics will be presented by randomized treatment group, and comparisons between study groups will be performed using the Student's t-test or Wilcoxon rank-sum test for continuous variables, and the chi-squared test or Fisher's exact test for categorical variables.

**10.3.2 Efficacy and safety endpoints**

The primary endpoint will be analyzed using the Cochran-Mantel-Haenszel method, stratified by study center, and expressed as odds ratio (OR) with 95% confidence intervals (CI) and associated *p*-values. For time-to-event endpoints, Kaplan-Meier survival curves and the log-rank test will be used to analyze and compare the time to the first event in each group. The reliability of all CI will be 95%, and the Cox proportional hazards regression model will be used to determine hazard ratios (HR). Categorical endpoints will be compared using chi-squared tests. This includes TIMI flow grade 3 post-PPCI, TMPG grade 3 post-PPCI, STR≥70% at 90 min after PPCI, and complete epicardial and myocardial reperfusion.

**10.4 Subgroup analyses**

For the primary endpoint, prespecified subgroup analyses were performed according to the following variables: gender, age, BMI, diabetes, Killip classification on admission, P2Y12 inhibitor use, the time interval from symptom onset to randomization, diabetes, systolic blood pressure on admission, annual PCI volume at each center. All subgroup analyses will require approval from the steering committee.

**10.5 Interim analysis**

No interim analysis will be carried out.

**11. Research quality management**

In compliance with Good Clinical Practice (GCP), clinical study monitors will be appointed to conduct regular monitoring visits. These visits will ensure strict adherence to all aspects of the study protocol and will include source data verification to confirm consistency with the content of the CRFs. During each monitoring visit, at a minimum, the following will be reviewed:

-Paper version of the ICF

-Adherence to the research protocol and its provisions

-Quality of data in the CRF: accuracy, missing data, consistency between data and source files (medical documents, appointment records, copies of laboratory test source reports, etc.)

**11.2 Independent Event Committee**

To ensure research quality, an independent CEC will conduct blind evaluations of all clinical endpoints at 30 days, 6 months, and 12 months post-randomization. The CEC will comprise experienced cardiologists and statistical experts who are not involved in the conduct of this study. CEC members will be blinded to randomized treatment assignments and all patient-identifying information. The CEC Charter will provide detailed definitions of all endpoint events, specifications for required source documents, and standardized procedures for the adjudication process.

**12. Ethics Committee**

An independent Ethics Committee will conduct a thorough review, approval, tracking, and periodic review of the final study protocol, including trial protocols, the final version of the ICF, and all other relevant documents. The Ethics Committee is responsible for safeguarding the rights and safety of study participants. The structure and activities of the Ethics Committee will be conducted independently, without any interference or influence from clinical trial organizations, sponsors, or investigators.

**Reference**

1. Byrne RA, Rossello X, Coughlan JJ, Barbato E, Berry C, Chieffo A, et al. 2023 ESC Guidelines for the management of acute coronary syndromes. *Eur Heart J* 2023;44:3720-3826. doi: 10.1093/eurheartj/ehad191.
2. Rakowski T, Dudek D, Dziewierz A, Yu J, Witzenbichler B, Guagliumi G, et al. Impact of infarct-related artery patency before primary PCI on outcome in patients with ST-segment elevation myocardial infarction: the HORIZONS-AMI trial. *EuroIntervention* 2013;8:1307-14. doi: 10.4244/EIJV8I11A199.
3. Emilsson OL, Bergman S, Mohammad MA, Olivecrona GK, Götberg M, Erlinge D, et al. Pretreatment with heparin in patients with ST-segment elevation myocardial infarction: a report from the Swedish Coronary Angiography and Angioplasty Registry (SCAAR). *EuroIntervention* 2022;18:709-718. doi: 10.4244/EIJ-D-22-00432.
4. Karlsson S, Andell P, Mohammad MA, Koul S, Olivecrona GK, James SK, et al. Editor's Choice- Heparin pre-treatment in patients with ST-segment elevation myocardial infarction and the risk of intracoronary thrombus and total vessel occlusion. Insights from the TASTE trial. *Eur Heart J Acute Cardiovasc Care* 2019;8:15-23. doi: 10.1177/2048872617727723.
5. Bloom JE, Andrew E, Nehme Z, Dinh DT, Fernando H, Shi WY, et al. Pre-hospital heparin use for ST-elevation myocardial infarction is safe and improves angiographic outcomes. *Eur Heart J Acute Cardiovasc Care* 2021;10:1140-1147. doi: 10.1093/ehjacc/zuab032.
6. Giralt T, Carrillo X, Rodriguez-Leor O, Fernandez-Nofrerias E, Rueda F, Serra-Flores J, et al. Time-dependent effects of unfractionated heparin in patients with ST-elevation myocardial infarction transferred for primary angioplasty. *Int J Cardiol* 2015;198:70-4. doi: 10.1016/j.ijcard.2015.06.009.
7. Giralt T, Ribas N, Freixa X, Sabaté M, Caldentey G, Tizón-Marcos H, et al. Impact of pre-angioplasty antithrombotic therapy administration on coronary reperfusion in ST-segment elevation myocardial infarction: Does time matter? *Int J Cardiol* 2021;325:9-15. doi: 10.1016/j.ijcard.2020.09.058.
8. Zijlstra F, Ernst N, de Boer MJ, Nibbering E, Suryapranata H, Hoorntje JC, et al. Influence of prehospital administration of aspirin and heparin on initial patency of the infarct-related artery in patients with acute ST elevation myocardial infarction. *J Am Coll Cardiol* 2002;39:1733-7. doi: 10.1016/s0735-1097(02)01856-9.
9. Fabris E, Menzio S, Gregorio C, Pezzato A, Stolfo D, Aleksova A, et al. Effect of prehospital treatment in STEMI patients undergoing primary PCI. *Catheter Cardiovasc Interv* 2022;99:1500-1508. doi: 10.1002/ccd.30153.
10. Verheugt FW, Liem A, Zijlstra F, Marsh RC, Veen G, Bronzwaer JG. High dose bolus heparin as initial therapy before primary angioplasty for acute myocardial infarction: results of the Heparin in Early Patency (HEAP) pilot study. *J Am Coll Cardiol* 1998;31:289-93. doi: 10.1016/s0735-1097(97)00495-6.
11. Chung WY, Han MJ, Cho YS, Kim KI, Chang HJ, Youn TJ, et al. Effects of the early administration of heparin in patients with ST-elevation myocardial infarction treated by primary angioplasty. *Circ J* 2007;71:862-7. doi: 10.1253/circj.71.862.
12. d'Entremont MA, Laferrière C, Bérubé S, Couture ÉL, Lepage S, Huynh T, et al. The effect of ASA, ticagrelor, and heparin in ST-segment myocardial infarction patients with prolonged transport times to primary percutaneous intervention. *Catheter Cardiovasc Interv* 2021;97:591-599. doi: 10.1002/ccd.29144.
13. Liem A, Zijlstra F, Ottervanger JP, Hoorntje JC, Suryapranata H, de Boer MJ, et al. High dose heparin as pretreatment for primary angioplasty in acute myocardial infarction: the Heparin in Early Patency (HEAP) randomized trial. *J Am Coll Cardiol* 2000;35:600-4. doi: 10.1016/s0735-1097(99)00597-5.
14. McGinley C, Mordi IR, Kell P, Currie P, Hutcheon S, Koch S, et al. Prehospital Administration of Unfractionated Heparin in ST-Segment Elevation Myocardial Infarction Is Associated With Improved Long-Term Survival. *J Cardiovasc Pharmacol* 2020;76:159-163. doi: 10.1097/FJC.0000000000000865.
15. Stähli BE, Varbella F, Linke A, Schwarz B, Felix SB, Seiffert M, et al. Timing of Complete Revascularization with Multivessel PCI for Myocardial Infarction. *N Engl J Med* 2023;389:1368-1379. doi: 10.1056/NEJMoa2307823.
16. Thiele H, Zeymer U, Neumann FJ, Ferenc M, Olbrich HG, Hausleiter J, et al. Intraaortic balloon support for myocardial infarction with cardiogenic shock. *N Engl J Med* 2012;367:1287-96. doi: 10.1056/NEJMoa1208410.
17. Montalescot G, van 't Hof AW, Lapostolle F, Silvain J, Lassen JF, Bolognese L, et al. Prehospital ticagrelor in ST-segment elevation myocardial infarction. *N Engl J Med* 2014;371:1016-27. doi: 10.1056/NEJMoa1407024.
18. Pu J, Ding S, Ge H, Han Y, Guo J, Lin R, et al. Efficacy and Safety of a Pharmaco-Invasive Strategy With Half-Dose Alteplase Versus Primary Angioplasty in ST-Segment-Elevation Myocardial Infarction: EARLY-MYO Trial (Early Routine Catheterization After Alteplase Fibrinolysis Versus Primary PCI in Acute ST-Segment-Elevation Myocardial Infarction). *Circulation* 2017; 136:1462-1473. doi: 10.1161/CIRCULATIONAHA.117.030582.

**IV. Supplementary Tables**

**Table S1 Clinical outcomes in PPS**

| **Outcomes** | **Cath Lab (n=480)** | **FMC (n=477)** | **HR (95% CI)** | ***P*-value** |
| --- | --- | --- | --- | --- |
| **At 30-day** |  |  |  |  |
| MACCE | 22/480 (4.6%) | 10/477 (2.1%) | 0.45 (0.23-0.91) | 0.034 |
| Total death | 14/480 (2.9%) | 10/477 (2.1%) | 0.72 (0.32-1.59) | 0.417 |
| Cardiac death | 14/480 (2.9%) | 10/477 (2.1%) | 0.72 (0.32-1.59) | 0.417 |
| Heart failure hospitalization | 5/480 (1.0%) | 0/477 (0.0%) | - | 0.025 |
| Reinfarction | 2/480 (0.4%) | 0/477 (0.0%) | - | 0.159 |
| Stent thrombus | 1/480 (0.2%) | 0/477 (0.0%) | - | 0.318 |
| Unplanned revascularization | 2/480 (0.4%) | 0/477 (0.0%) | - | 0.159 |
| Stroke | 1/480 (0.2%) | 0/477 (0.0%) | - | 0.315 |
| **At 180-day** |  |  |  |  |
| MACCE | 30/480 (6.3%) | 18/477 (3.8%) | 0.60 (0.34-1.05) | 0.079 |
| Total death | 15/480 (3.1%) | 14/477 (2.9%) | 0.93 (0.45-1.93) | 0.848 |
| Cardiac death | 15/480 (3.1%) | 14/477 (2.9%) | 0.93 (0.45-1.93) | 0.848 |
| Heart failure hospitalization | 9/480 (1.9%) | 2/477 (0.4%) | 0.22 (0.07-0.71) | 0.032 |
| Reinfarction | 1/480 (0.2%) | 0/477 (0.0%) | - | 0.159 |
| Stent thrombus | 2/480 (0.4%) | 0/477 (0.0%) | - | 0.318 |
| Unplanned revascularization | 4/480 (0.8%) | 1/477 (0.2%) | 0.25 (0.04-1.43) | 0.176 |
| Stroke | 2/480 (0.4%) | 1/477 (0.2%) | 0.49 (0.05-4.72) | 0.553 |
| **At 1-year** |  |  |  |  |
| MACCE | 32/480 (6.7%） | 25/477 (5.2%) | 0.77 (0.46-1.30) | 0.336 |
| Total death | 15/480 (3.1%) | 19/477 (4.0%) | 1.26 (0.64-2.47) | 0.501 |
| Cardiac death | 15/480 (3.1%) | 18/477 (3.8%) | 1.19 (0.60-2.36) | 0.61 |
| Heart failure hospitalization | 10/480 (2.1%) | 3/477 (0.6%) | 0.29 (0.10-0.87) | 0.048 |
| Reinfarction | 2/480 (0.4%) | 0/477 (0.0%) | - | 0.159 |
| Stent thrombus | 1/480 (0.2%) | 0/477 (0.0%) | - | 0.318 |
| Unplanned revascularization | 5/480 (1.0%) | 1/477 (0.2%) | 0.20 (0.04-0.98) | 0.099 |
| Stroke | 2/480 (0.4%) | 3/477 (0.6%) | 1.48 (0.26-8.52) | 0.668 |

Data are presented are n/N (%). PPS, per protocol set; CI, confidence interval; FMC, first medical contact; HR, hazard ratio; MACCE, major adverse cardiac and cerebrovascular event.

**Table S2 Clinical outcomes in PCIS**

| **Outcomes** | **Cath Lab (n=460)** | **FMC (n=473)** | **HR (95% CI)** | ***P*-value** |
| --- | --- | --- | --- | --- |
| **At 30-day** |  |  |  |  |
| MACCE | 23/460 (5.0%) | 10/473 (2.1%) | 0.42 (0.21-0.83) | 0.018 |
| Total death | 14/460 (3.0%) | 9/460 (1.9%) | 0.62 (0.27-1.41) | 0.260 |
| Cardiac death | 14/460 (3.0%) | 9/460 (1.9%) | 0.62 (0.27-1.41) | 0.260 |
| Heart failure hospitalization | 5/460 (1.1%) | 0/473 (0.0%) | - | 0.022 |
| Reinfarction | 2/460 (0.4%) | 0/473 (0.0%) | - | 0.151 |
| Stent thrombus | 1/460 (0.2%) | 0/473 (0.0%) | - | 0.309 |
| Unplanned revascularization | 3/460 (0.7%) | 0/473 (0.0%) | - | 0.079 |
| Stroke | 1/460 (0.2%) | 1/473 (0.2%) | 0.96 (0.06-15.34) | 0.976 |
| **At 180-day** |  |  |  |  |
| MACCE | 31/460 (6.7%) | 19/473 (4.0%) | 0.59 (0.34-1.02) | 0.063 |
| Total death | 15/460 (3.3%) | 13/473 (2.7%) | 0.83 (0.40-1.75) | 0.630 |
| Cardiac death | 15/460 (3.3%) | 13/473 (2.7%) | 0.83 (0.40-1.75) | 0.630 |
| Heart failure hospitalization | 9/460 (2.0%) | 3/473 (0.6%) | 0.32 (0.10-0.98) | 0.068 |
| Reinfarction | 2/460 (0.4%) | 0/473 (0.0%) | - | 0.151 |
| Stent thrombus | 1/460 (0.2%) | 0/473 (0.0%) | - | 0.309 |
| Unplanned revascularization | 5/460 (1.1%) | 1/473 (0.2%) | 0.19 (0.04-0.95) | 0.091 |
| Stroke | 2/460 (0.4%) | 2/460 (0.4%) | 0.95 (0.13-6.75) | 0.960 |
| **At 1-year** |  |  |  |  |
| MACCE | 33/460 (7.2%) | 26/473 (5.5%) | 0.75 (0.45-1.25) | 0.277 |
| Total death | 15/460 (3.3%) | 18/473 (3.8%) | 1.15 (0.58-2.28) | 0.685 |
| Cardiac death | 15/460 (3.3%) | 18/473 (3.8%) | 1.15 (0.58-2.28) | 0.685 |
| Heart failure hospitalization | 10/460 (2.2%) | 4/473 (0.8%) | 0.38 (0.13-1.08) | 0.088 |
| Reinfarction | 2/460 (0.4%) | 0/473 (0.0%) | - | 0.151 |
| Stent thrombus | 1/460 (0.2%) | 0/473 (0.0%) | - | 0.309 |
| Unplanned revascularization | 6/460 (1.3%) | 1/473 (0.2%) | 0.16 (0.04-0.70) | 0.051 |
| Stroke | 2/460 (0.4%) | 3/473 (0.6%) | 1.43 (0.25-8.25) | 0.695 |

Data are presented are n/N (%). PCIS, percutaneous coronary intervention set; CI, confidence interval; FMC, first medical contact; HR, hazard ratio; MACCE, major adverse cardiac and cerebrovascular event.

**Table S3 Safe endpoints in PPS**

|  | **Cath Lab (n=480)** | **FMC (n=477)** | **HR (95% CI)** | ***P*-value** |
| --- | --- | --- | --- | --- |
| **At 30-day** |  |  |  |  |
| BARC types 2-5 bleeding | 6/480 (1.3%) | 1/477 (0.2%) | 0.17 (0.04-0.74) | 0.059 |
| Access-site-related | 0/480 (0.0%) | 0/477 (0.0%) | - | - |
| Non-access-site-related | 6/480 (1.3%) | 1/477 (0.2%) | 0.17 (0.04-0.74) | 0.059 |
| Gastrointestinal | 4/480 (0.8%) | 1/477 (0.2%) | 0.25 (0.04-1.45) | 0.181 |
| intracranial | 1/480 (0.2%) | 0/477 (0.0%) | - | 0.319 |
| other | 1/480 (0.2%) | 0/477 (0.0%) | - | 0.317 |
| **At 180-day** |  |  |  |  |
| BARC types 2-5 bleeding | 10/480 (2.1%) | 6/477 (1.3%) | 0.60 (0.23-1.60) | 0.317 |
| Access-site-related | 0/480 (0.0%) | 0/477 (0.0%) | - | - |
| Non-access-site-related | 10/480 (2.1%) | 6/477 (1.3%) | 0.60(0.23-1.60) | 0.317 |
| Gastrointestinal | 6/480 (1.3%) | 4/477 (0.8%) | 0.67(0.19-2.30) | 0.528 |
| intracranial | 1/480 (0.2%) | 2/477 (0.4%) | 2.00 (0.21-19.21) | 0.564 |
| other | 3/480 (0.6%) | 0/477 (0.0%) | - | 0.083 |
| **At 1-year** |  |  |  |  |
| BARC types 2-5 bleeding | 10/480 (2.1%) | 6/477 (1.3%) | 0.60 (0.23-1.60) | 0.317 |
| Access-site-related | 0/480 (0.0%) | 0/477 (0.0%) | - | - |
| Non-access-site-related | 10/480 (2.1%) | 6/477 (1.3%) | 0.60 (0.23-1.60) | 0.317 |
| Gastrointestinal | 6/480 (1.3%) | 4/477 (0.8%) | 0.67 (0.19-2.30) | 0.528 |
| intracranial | 1/480 (0.2%) | 2/477 (0.4%) | 2.00 (0.21-19.21) | 0.564 |
| other | 3/480 (0.6%) | 0/477 (0.0%) | - | 0.083 |

Data are presented are n/N (%). PPS, per protocol set; FMC, first medical contact; HR, hazard ratio; CI, confidence interval; BARC, Bleeding Academic Research Consortium.

**Table S4 Safe endpoints in PCIS**

|  | **Cath Lab (n=460)** | **FMC (n=473)** | **HR (95% CI)** | ***P*-value** |
| --- | --- | --- | --- | --- |
| **At 30-day** |  |  |  |  |
| BARC types 2–5 bleeding | 6/460 (1.3%) | 2/473 (0.4%) | 0.32 (0.08-1.29) | 0.145 |
| Access-site-related | 0/460 (0.0%) | 0/473 (0.0%) | - | - |
| Non-access-site-related | 6/460 (1.3%) | 2/473 (0.4%) | 0.32 (0.08-1.29) | 0.145 |
| Gastrointestinal | 4/460 (0.9%) | 1/473 (0.2%) | 0.24 (0.04-1.40) | 0.169 |
| intracranial | 1/460 (0.2%) | 1/473 (0.2%) | 0.97 (0.06-15.55) | 0.984 |
| other | 1/460 (0.2%) | 0/473 (0.0%) | - | 0.309 |
| **At 180-day** |  |  |  |  |
| BARC types 2–5 bleeding | 10/460 (2.2%) | 6/473 (1.3%) | 0.58 (0.22-1.55) | 0.287 |
| Access-site-related | 0/460 (0.0%) | 0/473 (0.0%) | - | - |
| Non-access-site-related | 6/460 (1.3%) | 2/473 (0.4%) | 0.32 (0.08-1.29) | 0.145 |
| Gastrointestinal | 6/460 (1.3%) | 4/473 (0.8%) | 0.65 (0.19-2.23) | 0.494 |
| intracranial | 1/460 (0.2%) | 2/473 (0.4%) | 1.94 (0.20-18.65) | 0.582 |
| other | 3/460 (0.7%) | 0/473 (0.0%) | - | 0.078 |
| **At 1-year** |  |  |  |  |
| BARC types 2–5 bleeding | 10/460 (2.2%) | 6/473 (1.3%) | 0.58 (0.22-1.55) | 0.287 |
| Access-site-related | 0/460 (0.0%) | 0/473 (0.0%) | - | - |
| Non-access-site-related | 6/460 (1.3%) | 2/473 (0.4%) | 0.32 (0.08-1.29) | 0.145 |
| Gastrointestinal | 6/460 (1.3%) | 4/473 (0.8%) | 0.65 (0.19-2.23) | 0.494 |
| intracranial | 1/460 (0.2%) | 2/473 (0.4%) | 1.94 (0.20-18.65) | 0.582 |
| other | 3/460 (0.7%) | 0/473 (0.0%) | - | 0.078 |

Data are presented are n/N (%). PCIS, percutaneous coronary intervention set; FMC, first medical contact; HR, hazard ratio; CI, confidence interval; BARC, Bleeding Academic Research Consortium.

**V. Supplementary Figures**

**Figure S1**

**
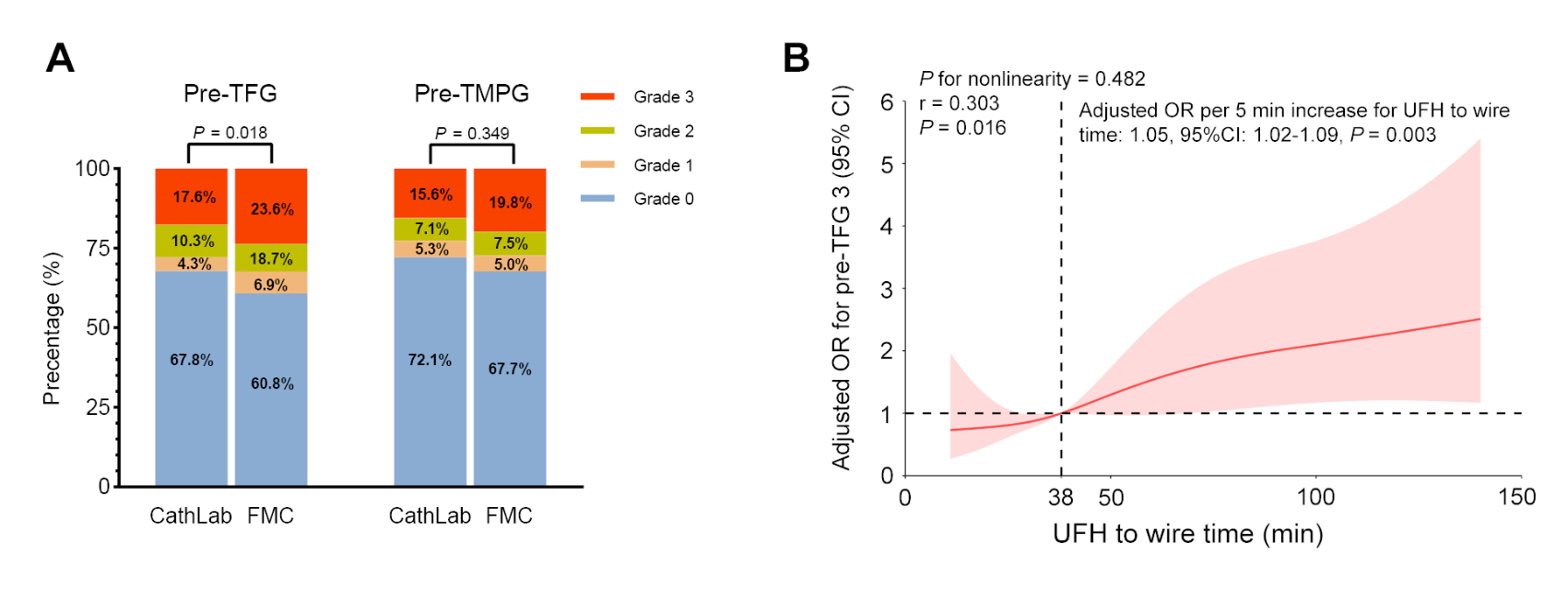
**

**Figure S2**

**
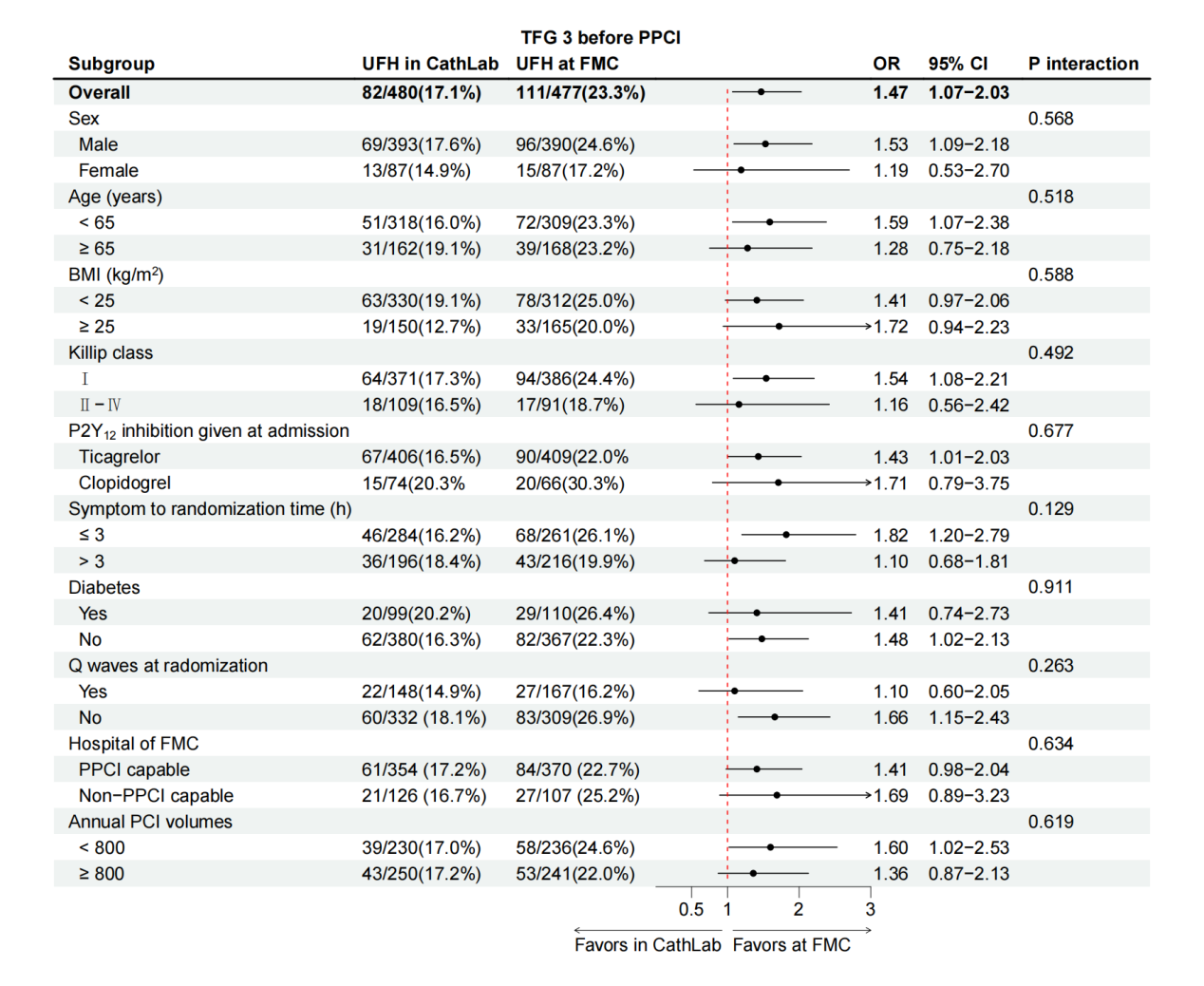
**

**Figure S3**

**
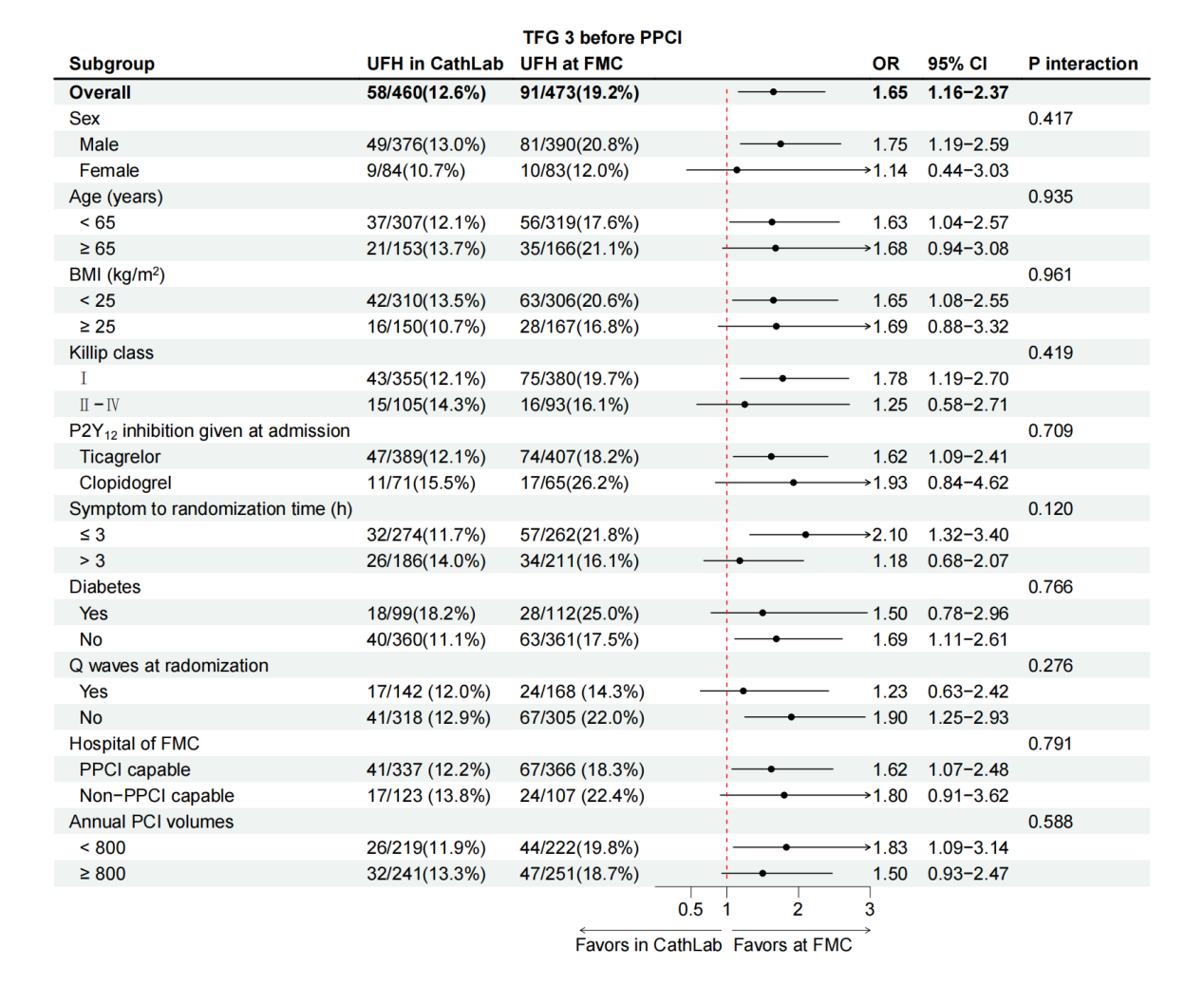
**

**Figure S4**

**Figure S4A**

**
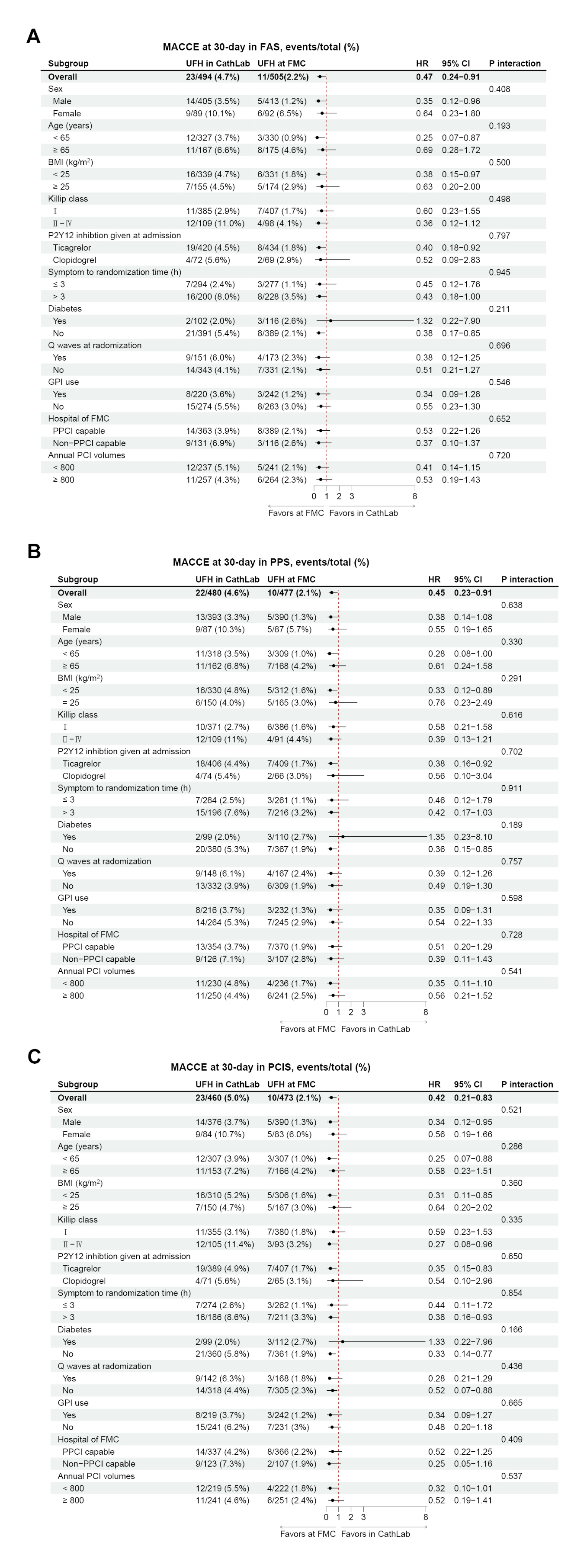
**

**Figure S4B**

**
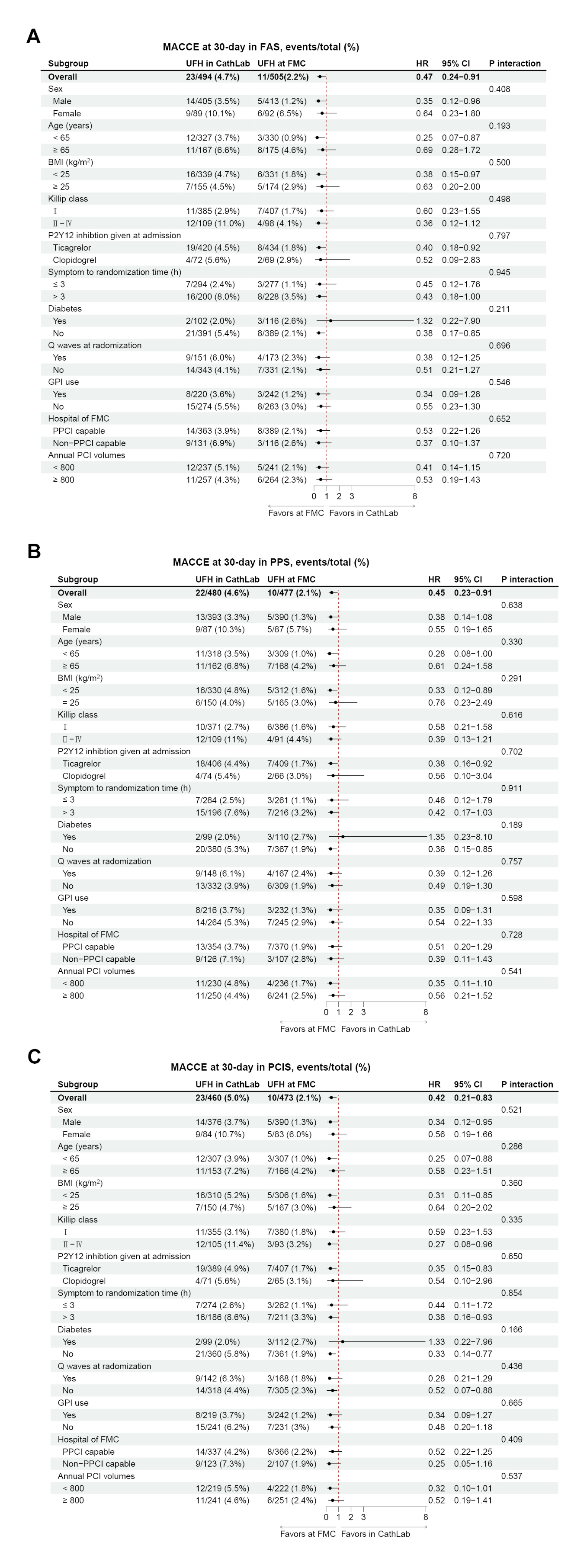
**

**Figure S4C**

**
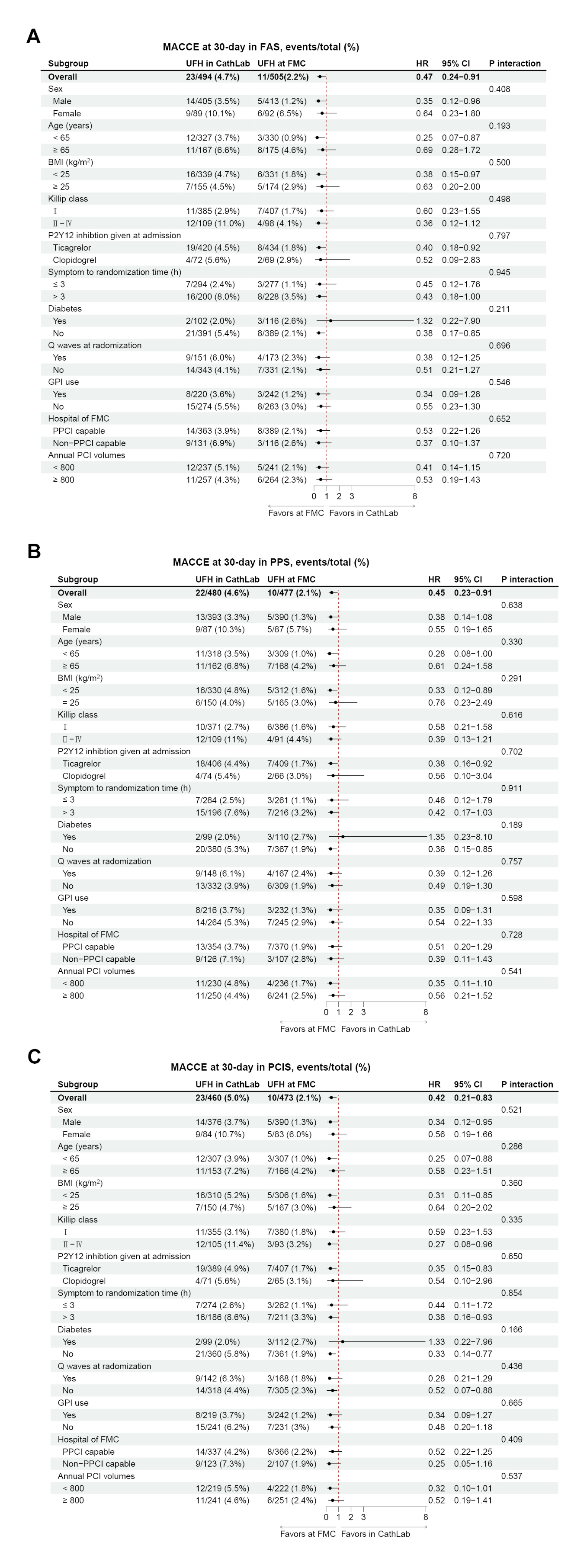
**

**Figure S5**

**Figure S5A**

**
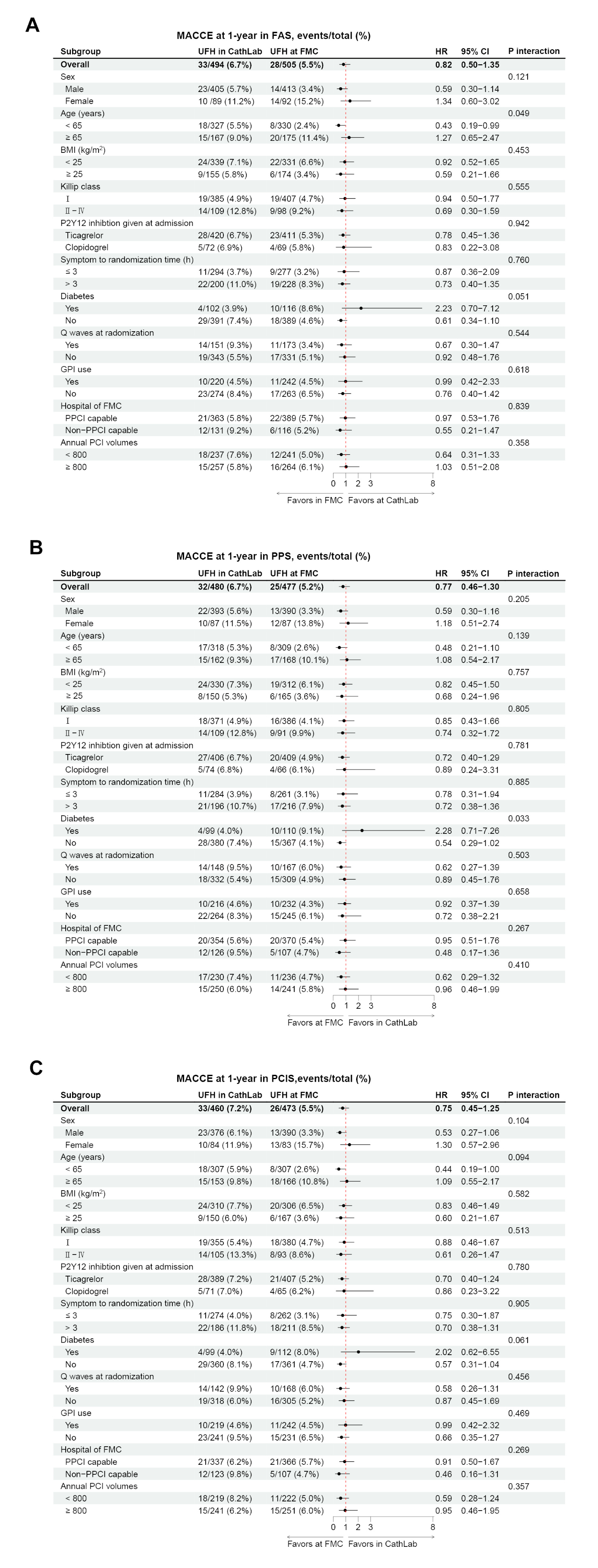
**

**Figure S5B**

**
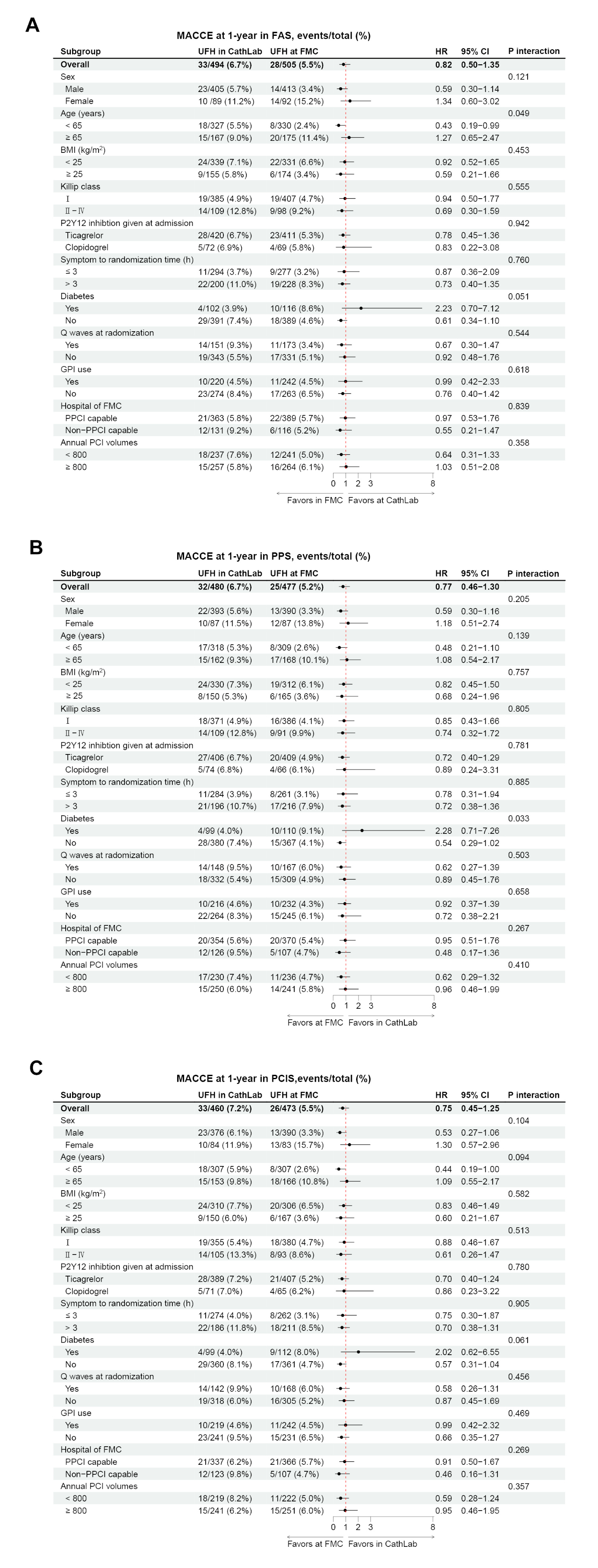
**

**Figure S5C**

**
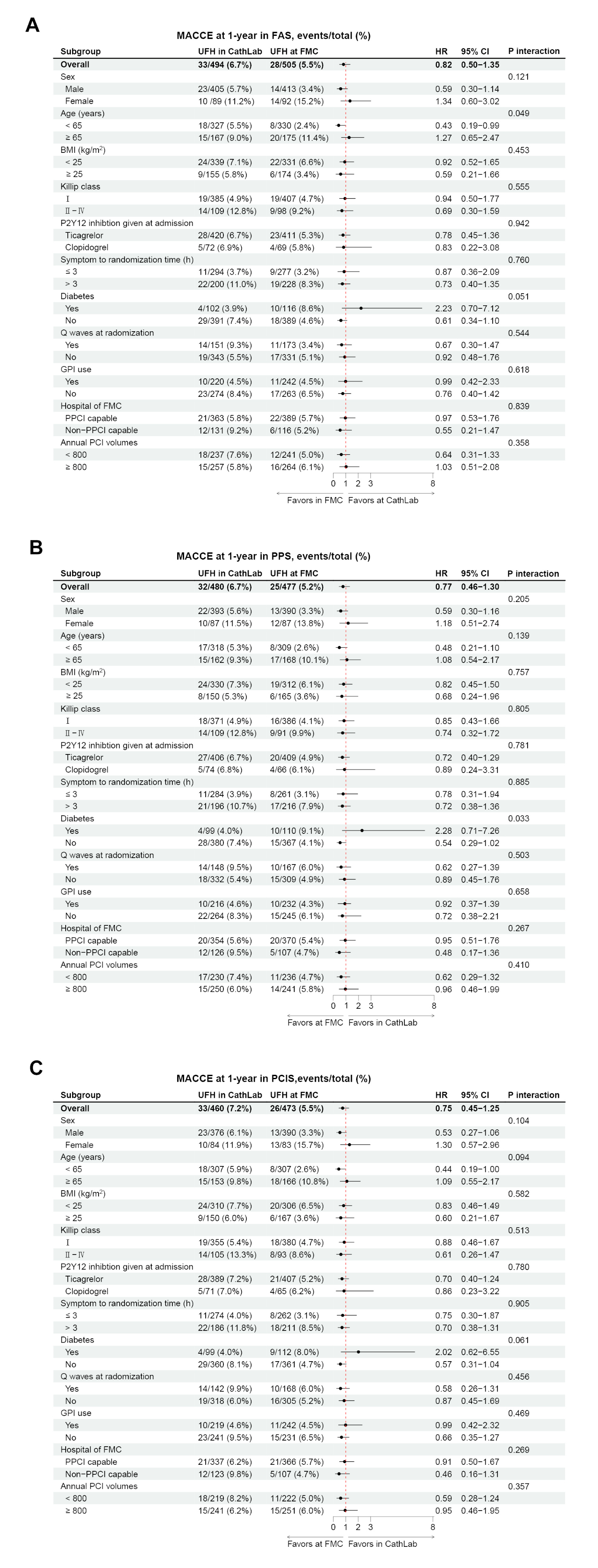
**

**Figure S6**

**
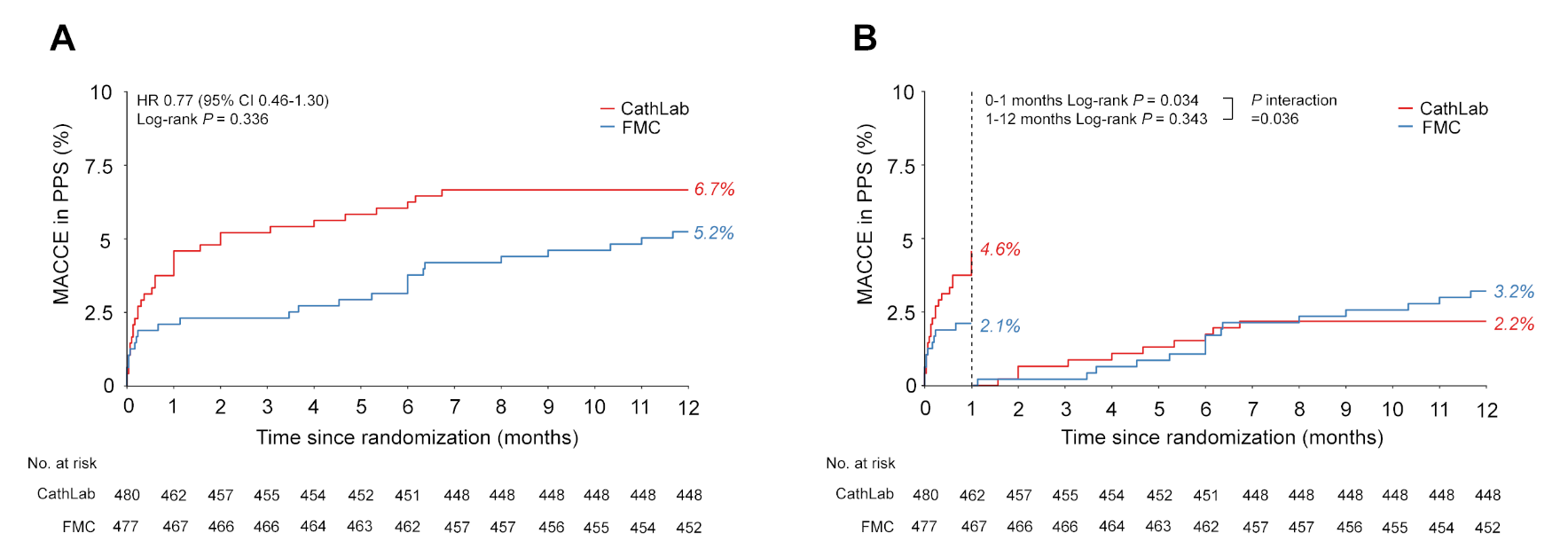
**

**Figure S7**

**
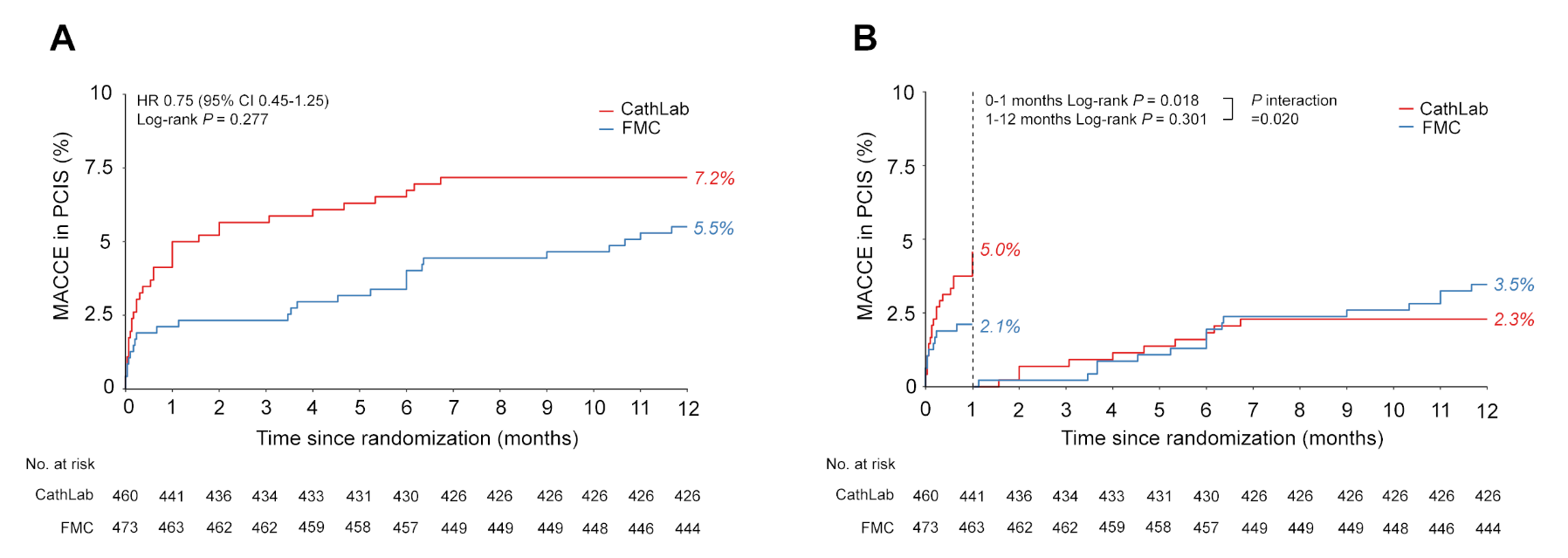
**

**Figure S8**

**
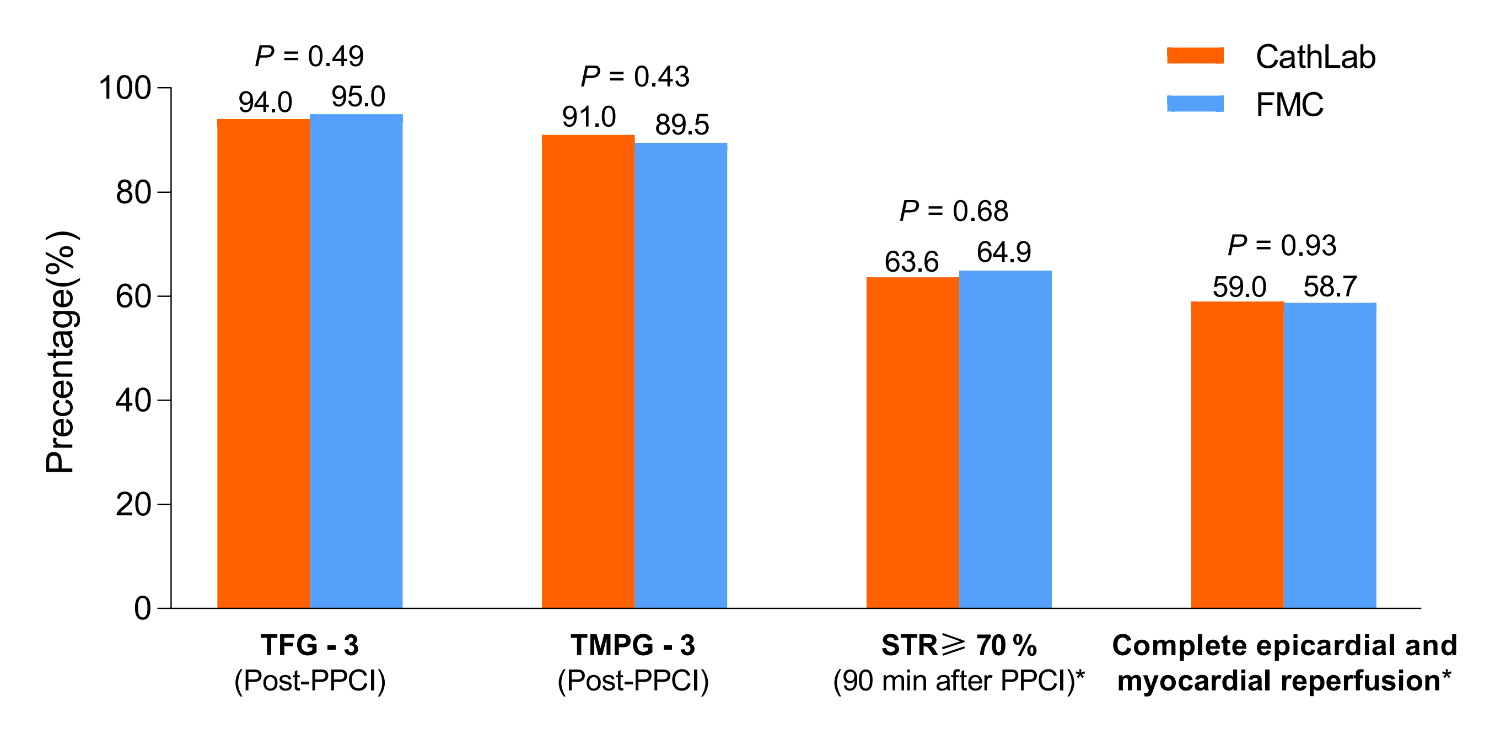
**

**Figure S9**

**
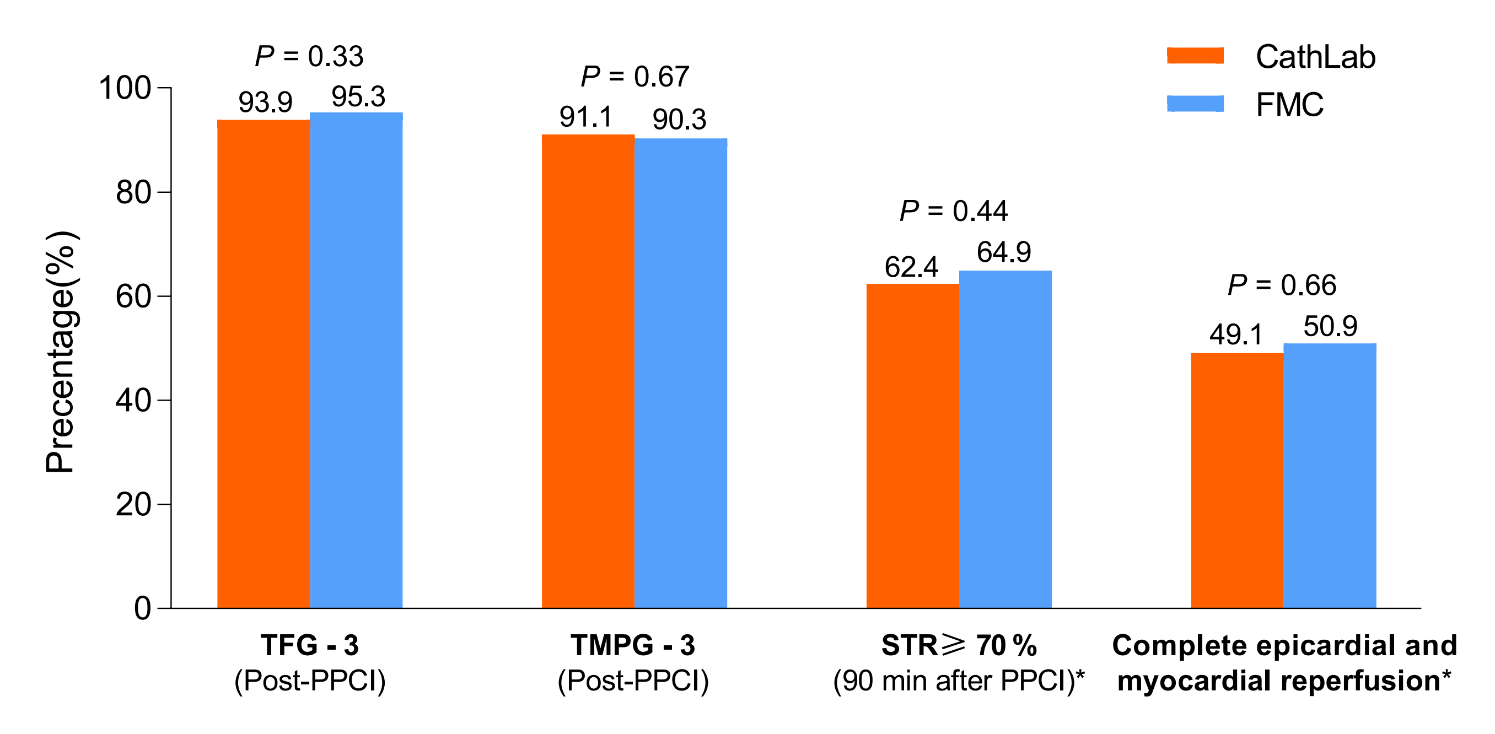
**

**Figure legends**

**Figure S1 A. Proportion of TFG and TMPG before PPCI in FAS. B. Restricted cubic splines for the association between pre-TFG 3 and UFH-to-wire time with** adjusted model. FAS, full analysis set. TIMI, thrombolysis in myocardial infarction; TMPG, TIMI myocardial perfusion grade; UFH, unfractionated heparin.

**Figure S2 Subgroup analyses of the primary endpoint among the 2 treatment arms in PPS.** PPS, Per Protocol Set; BMI, body mass index; CI, confidence interval; FMC, first medical contact; PCI, percutaneous coronary intervention; OR, odds ratio.

**Figure S3 Subgroup analyses of the primary endpoint among the 2 treatment arms in PCIS.** PCIS, percutaneous coronary intervention set; BMI, body mass index; CI, confidence interval; FMC, first medical contact; PCI, percutaneous coronary intervention; OR, odds ratio.

**Figure S4 Subgroup analyses of the 30-day MACCE in FAS (A), PPS (B) and PCIS (C).** FAS, full analysis set; PPS, Per Protocol Set; PCIS, percutaneous coronary intervention set; BMI, body mass index; CI, confidence interval; GPI, GP IIb/IIIa inhibitor; FMC, first medical contact; PCI, percutaneous coronary intervention; HR, hazard ratio.

**Figure S5 Subgroup analyses of the 1-year MACCE in FAS (A), PPS (B) and PCIS (C).** FAS, full analysis set; PPS, Per Protocol Set; PCIS, percutaneous coronary intervention set; BMI, body mass index; CI, confidence interval; GPI, GP IIb/IIIa inhibitor; FMC, first medical contact; PCI, percutaneous coronary intervention; HR, hazard ratio.

**Figure S6** **A.** **Kaplan-Meier curves for the 1-year MACCE in PPS. B. Time-to-event curves for MACCE with a landmark set at 30 days in PPS**. PPS, Per Protocol Set; CI, confidence interval; FMC, first medical contact; MACCE, major adverse cardiac and cerebrovascular event.

**Figure S7 A.** **Kaplan-Meier curves for the 1-year MACCE in PCIS. B. Time-to-event curves for MACCE with a landmark set at 30 days in PCIS.** PCIS, percutaneous coronary intervention set; CI, confidence interval; FMC, first medical contact; MACCE, major adverse cardiac and cerebrovascular event.

**Figure S8 Epicardial and myocardial reperfusion post-PPCI procedure among the 2 treatment arms in PPS.** PPS, Per Protocol Set; FMC, first medical contact; PPCI, primary percutaneous coronary intervention; TIMI, thrombolysis in myocardial infarction; TMPG, TIMI myocardial perfusion grade; STR, ST-segment resolution; *9 missing electrocardiogram data in PPS (2 in Cath Lab arm, 7 in FMC arm).

**Figure S9 Epicardial and myocardial reperfusion post-PPCI procedure among the 2 treatment arms in PCIS.** PCIS, percutaneous coronary intervention set; FMC, first medical contact; PPCI, primary percutaneous coronary intervention; TIMI, thrombolysis in myocardial infarction; TMPG, TIMI myocardial perfusion grade; STR, ST-segment resolution; *17 missing electrocardiogram data in PCIS (5 in Cath Lab arm, 12 in FMC arm).
